# Timing and dynamics of Late Wolstonian Substage ‘Moreton Stadial’ (MIS 6) glaciation in the English West Midlands, UK

**DOI:** 10.1098/rsos.220312

**Published:** 2022-06-29

**Authors:** Sebastian M. Gibson, Mark D. Bateman, Julian B. Murton, Timothy T. Barrows, L. Keith Fifield, Philip L. Gibbard

**Affiliations:** ^1^ Cambridge Quaternary, Department of Geography, University of Cambridge, Downing Place, Cambridge CB2 3EN, UK; ^2^ Department of Geography, University of Sheffield, Winter Street, Sheffield S10 2TN, UK; ^3^ Department of Geography, University of Sussex, Falmer, Brighton, BN1 9QJ, UK; ^4^ School of Earth, Atmospheric and Life Sciences, University of Wollongong, Australia; ^5^ School of the Environment, Geography and Geosciences, University of Portsmouth, UK; ^6^ Department of Nuclear Physics, Research School of Physical Sciences and Engineering, The Australian National University, Canberra, ACT 0200, Australia; ^7^ Scott Polar Research Institute, University of Cambridge, Lensfield Road, Cambridge CB2 1ER, UK

**Keywords:** Quaternary, Wolstonian Stage, lithostratigraphy, sedimentology, optically stimulated luminescence, exposure dating

## Abstract

Glaciation during the late Middle Pleistocene is widely recognized across continental northwest Europe, but its extent and palaeoenvironmental significance in the British Isles are disputed. Although glaciogenic sediments at Wolston, Warwickshire, in the English West Midlands, have been used to define the stratotype of the Wolstonian Stage, their age has been variably assigned between marine isotope stages (MIS) 12 and 6. Here we present sedimentological and stratigraphical observations from five sites across the English West Midlands whose chronology is constrained by new luminescence ages from glaciofluvial sediments, supplemented by cosmogenic ^36^Cl exposure dating of erratic boulders. The ages suggest that between 199 ± 5 and 147 ± 2.5 ka the British Ice Sheet advanced into the English West Midlands as far south as Moreton-in-Marsh, Gloucestershire. This advance is assigned to the Moreton Stadial of the Late Wolstonian Substage. Dating of the glaciation to this substage allows correlation of the Moreton Stadial glacial deposits in the English West Midlands with those of the Drenthe Stadial during the Late Saalian Substage across continental northwest Europe.

## 1. Introduction

**Figure 1.**
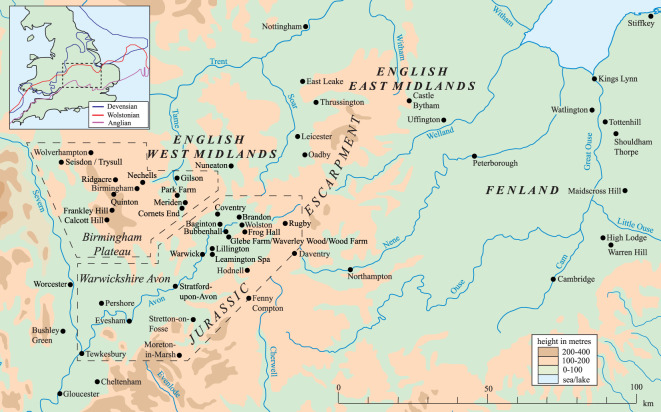
Map of the topography and location of the English West Midlands (Birmingham, Severn, Warwickshire Avon and Jurassic Escarpment), East Midlands and the Fenland Basin. The inse t map summarizes previously published limits of glaciation during the Anglian, Wolstonian and Devensian stages in the southern British Isles [2].

The onshore Quaternary record of the British Isles contains geological evidence for at least three lowland glaciations during the Middle Pleistocene and Late Pleistocene: the Anglian (*ca* Marine Isotope Stage (MIS) 12), Wolstonian (*ca* MIS 6), and Devensian (*ca* MIS 2) stages. Evidence for the second glaciation—the Wolstonian—was identified by Shotton [1] from a complex sequence of glacial and fluvial sediments in the English West Midlands (figure 1, table 1). The uncertainty of the palaeoenvironmental history and the age of the Pleistocene sequences in the English West Midlands relative to those in the English East Midlands and Fenland Basin developed since the 1980s—to the extent that it became widely believed that the English West Midlands sequences should be re-assigned to the older, Anglian Stage glaciation [20,22–24]. The extent of glaciation in the English West Midlands, and the complexity of the sequences more generally across the British Isles, has raised questions as to the timing and extent of glaciations, the development of the drainage throughout the Pleistocene and how the palaeoenvironmental history in the region correlates with that in other regions of the British Isles and continental Europe [25].

**Table 1.** Description and interpretation of genesis and age of cold-climate sedimentary deposits of the English West Midlands. At each site, the oldest lithostratigraphical unit is at the bottom of the description and the youngest is at the top.

site	location	description	lithostratigraphy	interpretation	stage	author(s)
Baginton (Baginton Hill)	SP339750	Gravelly CLAY (Diamicton). With Mercia Mudstone, Bunter pebbles, quartzite, quartz and occasional sandstone erratics. 1.2 m thick.	Thrussington Till	Glacial Till	Wolstonian	Shotton [1,3]
		Fine red SAND. With layers of Bunter pebbles. 3.65 m thick.	Baginton Sand	Glaciofluvial		
		Coarse GRAVEL. With Bunter pebbles. 3.0 m thick	Baginton-Lillington Gravel	Glaciofluvial		
Brandon (Pools Farm)	SP 348750	SAND and GRAVEL. Upward fining, horizontally bedded, clast-supported gravel and massive sand. With Bunter pebbles, sandstone and chert. Ice-wedge pseudomorph and involutions extending through an erosional, planar contact into underlying unit. 1.55 m thick.	Upper Brandon Sand and Gravels	Avon 4th Terrace	Wolstonian (Devensian periglacial)	Shotton [1,4], Maddy [5]
		Organic, laminated SILT and CLAY. Cryoturbated. Small to medium Bunter clasts. Infilled channel, erosional, concave-up into underlying unit. 1.8 m thick.	Upper Brandon Organic Silts and Clay			
		Fine to medium SAND. Trough and planar cross-stratified bedding. Lenses of clast-supported gravel with interbedded massive sand up to 0.5 m thick. Palaeocurrent to northeast. Erosional plane with underlying unit. 6.5 m thick.	Baginton Sand	Glaciofluvial/fluvial	Wolstonian	
		Organic SAND and SILT. Sand is massive, silt is laminated. Occasional lenses of massive fine to medium sandy gravel. Boundary is erosional, concave-upward to underlying unit. 2.1 m thick.	Lower Brandon Organic Sand and Silts			
		Sandy GRAVEL. Clast-supported, massive, medium to coarse. With Bunter pebbles, quartz, quartzite, Mercia Mudstone and chert/sandstone. Occasional sub-horizontal bedding in sand lenses. 3.5 m thick.	Baginton-Lillington Gravel	Glaciofluvial/fluvial		
		Sandy GRAVEL. Matrix-supported, cryoturbated. 2.5 m thick Laminated silty SAND. Horizontal bedding. Occasional fine to medium Bunter pebbles and coal. 1.4 m high ice-wedge pseudomorph. 4 m thick.				
		Gravelly SAND. Coarse sand with fine to medium gravel bedding with Bunter pebbles. 3.5 m thick				
		SAND and GRAVEL. Sand cross-bedded. Coarse gravel with Bunter pebbles, sandstone, granite, rhyolite, andesite and coal. 12 m thick.				
Cornets End	SP233811	Sandy GRAVEL. Matrix-supported, cryoturbated. 2.5 m thick Laminated silty SAND. Horizontal bedding. Occasional fine to medium Bunter pebbles and coal. 1.4 m high ice-wedge pseudomorph. 4 m thick.	Cornets End Upper Gravel	Solifluction lobe	Devensian/Wolstonian	Shotton [6], Brown [7]
			Cornets End Silty Sand/Wolston Clay	Glaciolacustrine	Wolstonian	
		Gravelly SAND. Coarse sand with fine to medium gravel bedding with Bunter pebbles. 3.5 m thick	Cornets End Sand/ Baginton Sand	Glaciofluvial		
		SAND and GRAVEL. Sand cross-bedded. Coarse gravel with Bunter pebbles, sandstone, granite, rhyolite, andesite and coal. 12 m thick.	Cornets End Lower Gravel/Baginton-Lillington Gravel	Glaciofluvial		
Frankley	SO992804	Silty gravelly CLAY (Diamicton). Unknown thickness on hill plateaus around Frankley. With angular North Wales originated quartz, quartzite, sandstone, coal and Mercia Mudstone.	Frankley Hill Till	Glacial Till	Wolstonian	Crosskey [8], Harrison [9]
Frog Hall 1950 s	SP415738	Sandy GRAVEL. Sand is interbedded within the gravel. With flint, Bunter pebbles, ironstone and sandstone. 6 m thick.	Dunsmore Gravel	Glaciofluvial/Avon 5th Terrace	Wolstonian	Shotton [1]
Frog Hall 1980 s	SP415736	Clayey sandy GRAVEL. Poorly sorted, with angular to sub-angular flint, rounded Bunter pebbles, sandstone and ironstone. 4 m to 4.5 m in thickness.	Dunsmore Gravel	Glaciofluvial/Avon 5th Terrace	Wolstonian	Sumbler [10], Old *et al.* [11]
		Reported, but undescribed CLAY. Thickness unknown. Surface difference between upper unit and lower *ca* 10 m.	Wolston Clay	Glaciolacustrine		
		Clayey SAND and GRAVEL. With flint, Bunter pebbles, ironstone, and sandstone. 9 m thick.	Frog Hall Sand and Gravel	Glaciofluvial/Avon 4th Terrace	Wolstonian/Ipswichian	
Frog Hall 1990 s	SP415734	Clayey sandy diamicton with flint, chert, quartzite and quartz. 3 m thick.	Frog Hall Diamicton	Glacial Till	Wolstonian	Keen *et al.* [12]
		Sandy GRAVEL. Horizontal bedded gravel and planar cross-stratified sand. Palaeocurrent to south west. With flint, ironstone, quartzite, quartz and chert 2.10 to 5.40 m thick.	Upper Frog Hall Sand and Gravel	Fluvial/Avon 4th Terrace		
		Organic SILT (‘mud’). Interbedded. 7.40 m thick.	Frog Hall Silt	Fluvial		
		Sandy GRAVEL. With limestone, flint, ironstone, quartzite and quartz 4.0 to 6.50 m thick.	Lower Frog Hall Sand and Gravel			
Hodnell	SP422570	Sandy GRAVEL. Coarse with Bunter pebbles, flint, ironstone, limestone and sandstone. 2.7 m thick.	Dunsmore Gravel	Glaciofluvial/Avon 5th Terrace	Wolstonian	Bishop [13]
		Silty CLAY. Calcareous. Stoneless orange silt and grey-purplish clay. 1.5 m thick.	Wolston Clay	Glaciolacustrine		
		Gravelly SAND. Medium to coarse, massive, with occasional lenses of Bunter pebbles. 1.2 m thick.	Wolston Sand and Gravel	Glaciolacustrine/Glaciofluvial		
		Silty gravelly CLAY (Diamicton). Grey-brown, calcareous with chalk, flint and Bunter pebbles. 7.0 m thick.	Hodnell Clay	Glacial Till/Glaciofluvial		
Leamington Spa (Cubbington Church)	SP341681	Gravelly CLAY (Diamicton). With Mercia Mudstone, Bunter pebbles, quartzite, quartz and occasional sandstone erratics. 1.5 m thick.	Thrussington Till	Glacial Till	Wolstonian	Shotton [3]
		Fine brown SAND. With layers of fine Bunter pebbles. Irregular bedding 3.0 m thick.	Baginton Sand	Glaciofluvial/fluvial		
		Sandy GRAVEL. ‘uneven bedding’. Gravel is fine with Bunter pebbles, quartzite, quartz, and sandstone. 0.9 m thick	Baginton-Lillington Gravel	Glaciofluvial/fluvial		
Leamington Spa (Radford Semele Church)	SP350644	Gravelly CLAY (Diamicton). Red–blue with chalk, flint, limestone, dolerite, quartzite, quartz and Mercia Mudstone. 2.75 m thick.	Oadby Till	Glacial Till	Wolstonian	Shotton [1,3]
		Laminated CLAY. Interbedded with SAND and GRAVEL. Coarse sand with Bunter pebbles, quartz and coal. 2.75 m thick.	Wolston Clay and Wolston Sand and Gravel	Glaciolacustrine/Glaciofluvial		
Lillington (Pratt's Pit)	SP330672	Gravelly CLAY (Diamicton). With Mercia Mudstone, Bunter pebbles, quartzite, quartz and occasional sandstone erratics. 1.5 m thick.	Thrussington Till	Glacial Till	Wolstonian	Shotton [1,3]
		Fine brown SAND. With layers of fine Bunter pebbles. Irregular bedding 3. m thick.	Baginton Sand	Glaciofluvial/fluvial		
		Sandy GRAVEL. ‘uneven bedding’. Gravel is fine with Bunter pebbles, quartzite, quartz, sandstone and flint. 2.1 m thick	Baginton-Lillington Gravel	Glaciofluvial/fluvial		
Meriden	SP235823	Sandy gravelly CLAY (Diamicton). Reddish-brown, stiff. Sand fine to medium. Gravel fine to medium, sub-angular to sub-rounded. Between 1.0 m and 7.0 m.	Thrussington Till	Glacial Till	Wolstonian	Cannell [14]
Nechells	SP094892	Clayey SAND and GRAVEL. Gravel is fine to coarse in a medium to coarse clayey sand. Fabric orientation north-north east to south-south east. 5.5 m thick.	Nechells Upper Glacial Series	Glaciofluvial	Wolstonian	Kelly [15]
		A series of lake clays (c. 2.1 m), formed of: finely laminated silts and fine sands (0.3 m), overlain by varved clay (1.3 m) and varved clay with interbedded silty fine sand (0.7 m).	Nechells Late-glacial Lake Series	Glaciolacustrine		
		A series of basal coarse gravels (c. 22.8 m), sand and fine gravel (c. 3.6 m). The sand is horizontally bedded with silt, fine sand. Bedding thickness between 3 cm and 30 cm, with an average 10 cm thickness. Gravel contains Coal. Basal sediments are overlain by interbedded silt and massive medium sand (c. 11.6 m) and a medium to coarse sand with gravel, coarsening upwards (c. 10 m).	Nechells Lower Glacial Series	Glaciofluvial	Anglian	
Stretton-on-Fosse Pit	SP218382	Gravelly CLAY (Diamicton). Faulted. Grey, with chalk, Bunter pebbles, siltstone, flint, limestone, ironstone, chert and quartzite. 2.1 m thick	Moreton Till	Glacial Moraine	Wolstonian	Tomlinson [16], Bishop [13]
		Gravelly CLAY. Laminated. Brown-red. Occasional lenses of sand with Bunter pebbles. 0.9 m thick.	Wolston Clay	Glaciolacustrine		
		GRAVEL. Crude bedding. With sub-rounded oolite, ironstone and Bunter pebbles. Base of unit false-bedded clayey gravel. 2.4 m thick.	Paxford Gravel	Glaciofluvial		
		Gravelly SAND. False-bedded with lenses of quartzite and Bunter pebbles. Rare flints. 9.0 m thick.	Stretton Sands	Glaciofluvial		
Trysull	SO845949	SAND and GRAVEL. Fine to coarse sand with top-set, fore-set and bottom-set bedding. Sandy matrix-supported gravel with sandstone, Bunter pebbles, white and pink granite, felsites and andesites. Sandstone is well-rounded, average 1 m by 0.5 m size. Structure is tilted with faulting. 27.2 m thick.	Trysull Sand and Gravel	Glaciofluvial	Wolstonian	Morgan [17]
Waverley Wood	SP365715	Silty CLAY (Diamicton). 1 m thick. Contains Mercia Mudstone, sandstone, and coal.	Thrussington Till	Glacial Till	Wolstonian	Shotton *et al.* [18]
		Coarse SAND. Cross-bedding with occasional Bunter pebbles.	Baginton Sand	Glaciofluvial/fluvial		
		Sandy GRAVEL. Fine to medium with Bunter pebbles quartzite and quartz. 8 m thick.	Baginton-Lillington Gravel	Glaciofluvial/fluvial		
		Sandy SILT overlying the Mercia Mudstone. Maximum thickness 2 m. Five distinct channels within unit of: 1) a silty sand with quartzite pebbles 0.2 m thick; 2) organic ‘mud’ with plant and shell detritus 0.6 m in thickness; 3) organic silty sand 0.2 m in thickness; 4) organic ‘mud’ with quartzite pebbles 0.15 m in thickness, and 5) organic ‘mud’ with plant and shell detritus 0.45 m in thickness.	Waverley Wood Silt and Sand	Fluvial	early Wolstonian (Cromerian?)	
Wood Farm	SP370723	Silty CLAY (Diamicton). 3 m thick. Contains Mercia Mudstone, sandstone, quartzite and coal.	Thrussington Till	Glacial Till	Wolstonian	Keen *et al.* [19]
		Medium SAND. 2 m thick. Well sorted cross-bedding to north east with occasional fine Bunter pebbles.	Baginton Sand	Glaciofluvial		
		Sandy GRAVEL. Medium with upward grading to fine with Bunter pebbles quartzite. 2 m thick.	Baginton-Lillington Gravel	Glaciofluvial/fluvial		
		Silty gravelly SAND. With organic detritus. Gravel is fine. 1.8 m thick.	Waverley Wood Silt and Sand	Fluvial	early Wolstonian (Cromerian?)	
Wolston	SP410746	Sandy GRAVEL with Bunter pebbles and flint	Dunsmore Gravel	Glaciofluvial	Wolstonian	Shotton [1], Lewis [20]
		Occasionally laminated, massive silty CLAY. 7 m thick. Calcareous. Occasional clasts of Mercia Mudstone and chalk.	Upper Wolston Clay	Glaciolacustrine		
		Gravelly SAND. 6.1 m thick	Wolston Sand	Glaciofluvial		
		Massive clayey SILT becoming a laminated CLAY. Variable thickness from 2.5 to 3.6 m. Occasional clasts of Mercia Mudstone and fine Bunter pebbles, sandstone, and coal.	Lower Wolston Clay	Glaciolacustrine		
		Occasionally laminated silty sandy CLAY (Diamicton). 4 m thick. Poorly sorted, massive. Contains Bunter pebbles, quartzite, quartz, limestone, siltstone, and coal.	Thrussington Till	Glacial Till		
		Fine to medium, well sorted SAND. Ripple bedding rising into sub-horizontal bedding. Variable thickness from 0.5 m to 4.8 m	Baginton Sand	Glaciofluvial/fluvial		
		Sandy GRAVEL.3 m thick	Baginton-Lillington Gravel	Glaciofluvial/fluvial		
Quinton	SO992847	Sandy gravelly CLAY (Diamicton). Bedded, red–brown and orange becoming unbedded (massive) with Triassic and Carboniferous erratics. 2.5 m to 5 m thick.	Ridgeacre Till	Glacial Till	Wolstonian	Horton [21]
		Purplish brown sandy gravelly CLAY with erratics of local origin from the Coal Measures. Some sections demonstrated local lamination of the clay. With basal clay being red, becoming purplish.	Nurseries Till	Glacial Till	Anglian	

**Figure 2.**
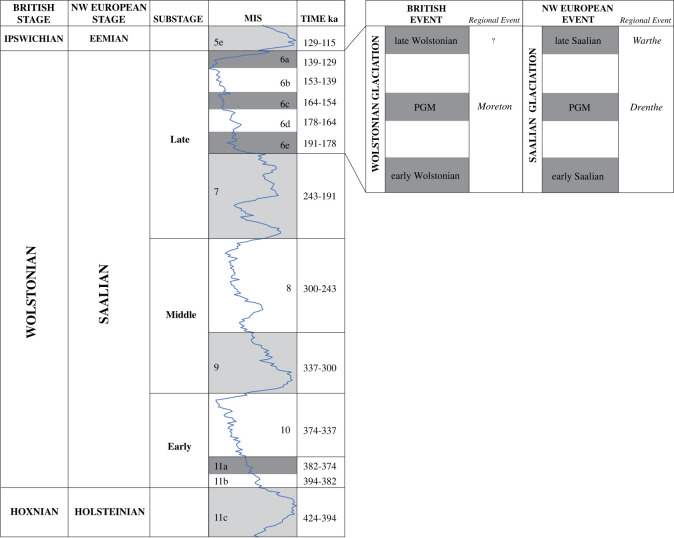
Chronostratigraphical division and correlation of the regional Middle Pleistocene stages and corresponding events in the English West Midlands, East Anglia and the continental record (compiled from [2,28,29,31,32,34,40]). The Wolstonian Stage is defined between *ca* MIS 11b and *ca* MIS 5e by Shotton [1,6,36], Litt & Turner [27] and Gibbard & Turner [39]. Subdivision of *ca* MIS 6 into sub-stages 6a to 6e (time in ka) follows Sun & An [30].

The formal equivalent MIS of the Wolstonian Stage is defined as that from *ca* MIS 11b to 6e (cf. [1,6,26–32]. This represents the period of time between the termination of the Hoxnian Interglacial Stage [33,34] and the initiation of the Ipswichian Interglacial Stage (cf. [35]) [1,6,26,27,36–39]. Figure 2 represents the Wolstonian Stage (and its equivalent Saalian Stage in continental northwest Europe) as the time interval of approximately 260 000 years [31]. The division of the Wolstonian Stage has been effectively achieved from the informal division based on the Early, Middle and Late Substages, which equate broadly with the marine isotope stratigraphical record [31,32,40,41]. It is important to note that the terrestrial environment responded differently to the oceans, with the chronostratigraphic stages and substages correlated with climate-driven event stratigraphy [31]. The marine isotope stratigraphy glacial maximum of the Wolstonian Stage (Saalian: NW Europe; Moscovian/Dnieper: Russian Plain and Illinoian: N America) occurred within MIS 6a at 140 ka [31,41], whereas there is much evidence for the terrestrial Wolstonian Stage in continental Europe (Saalian) occurring earlier in *ca* MIS 6c at 160 ka [2,31,40,42–48].

**Figure 3.**
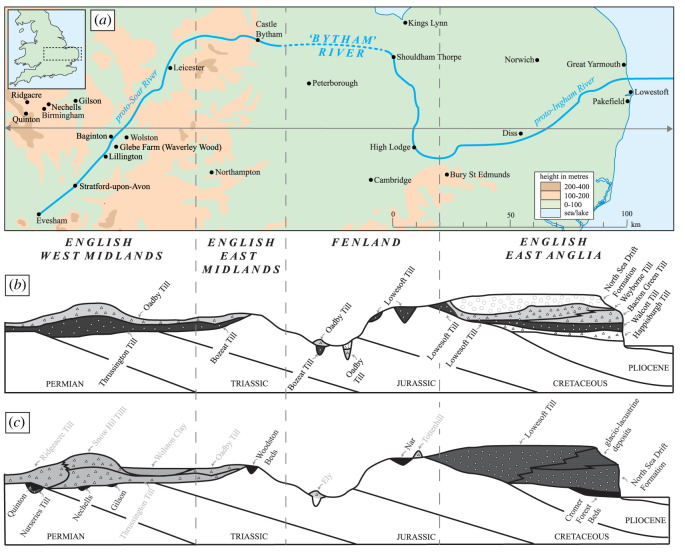
Map of the proposed route of the ‘Bytham River’ modified after Rose [23,51], Lewis [20] and Lee *et al.* [24,52] and a schematic cross-section across the route showing the main glacial event stratigraphy deposited during the Wolstonian Stage (light grey) and Anglian Stage (dark grey) modified from Lee *et al.* [52]. (*a*) The dashed line on the map shows Rose [51] and Lewis's [20] proposed Fenland Basin route. Separately, to the west is the proto-Soar River flowing northeastwards, modified from Shotton [1,26]. To the east is the ancient Ingham River. These two rivers are regarded as diachronous in the present study, although they transported similar (common) lithologies. (*b*) The revised stratigraphy for southern Britain proposed by Lee *et al.* [24,52], based on the occurrence of a ‘Bytham River’ [22], which has been discounted in Gibbard *et al.* [53]. (*c*) The ‘classical’ regional stratigraphy, distinguishing between Wolstonian and Anglian glacial stages, based on regional event stratigraphy from Shotton [1] and Ehlers *et al.* [54].

The proto-Soar River, which deposited the quartz-rich (*Bunter pebbles*) gravel (Baginton–Lillington Gravel Member) and sands (Baginton Sand Member) in the Baginton Formation, was thought by Shotton [1] to have formed shortly after the Hoxnian Interglacial Stage and persisted until it was overridden by advancing Wolstonian Stage ice which deposited the Wolston ‘*Series’*, now termed the Wolston Glacigenic Formation [49]. The problem with this observation is that the Wolstonian-type ‘*Bunter pebble*’ deposits are neither underlain nor overlain by interglacial sediments of the Hoxnian or Ipswichian age deposits (figure 2) [25]. Rose [22,23,50,51], however, attributed the Baginton Formation sand and gravels to deposition by the headwaters of a pre-Anglian Stage ‘*Bytham*’ River (figure 3). This interpretation was based on the correlation of quartz-rich gravel through and from the English East Midlands site of Castle Bytham, Lincolnshire (SK 980 187; figures 1 and 3), and equated to the deposits of the proto-Ingham River in central East Anglia [56–58] (figure 3). The quartz-rich Ingham Formation gravels underlie the Lowestoft Formation Till of the Anglian Stage in East Anglia. Thus the ‘*Bytham*’ River was thought to be aligned from Breedon Hill in Warwickshire toward Pakefield in Suffolk, across the Jurassic escarpment at Castle Bytham, Lincolnshire and the Fenland Basin (figure 3). Rose [22,23,50,51,59], Lewis [20] and Lee *et al.* [24,52] have attributed the overlying Wolston Formation to the Anglian glaciation. Furthermore, at Red Barn Quarry, Castle Bytham, the glaciofluvial sediments are overlain by glacial sediments correlated to the Oadby Till Member of the Wolstonian Stage [52]. Overall, therefore, the nub of the Wolstonian ‘problem’ [25] is that it is possible to correlate the sequence of both lithostratigraphical models to that of the Anglian Stage (*ca* MIS 12) [20,22–24,50–52,59] or that of the Wolstonian Stage (*ca* MIS 6) [1,4,6,26,36,60,61]. Consideration of the Wragby Till Member of Lincolnshire bears some relevance to understanding the timing of glaciation during the Wolstonian Stage, since it has been considered to be an equivalent of the Oadby Till [62–64], despite the advance across Lincolnshire having been correlated to MIS 8 [65,66].

**Table 2.** Luminescence dating data and ages.

site	sample lab. code	stratigraphic information	depth (m)	quartz/ feldspar	total dose rate (μGy/ka)	measurement^a^	*n*^b^	saturated	*D*_e_ (Gy)	proportion^d^ (%)	OD (%)	age^e^ (ka)
Meriden	Shfd15022	Meriden Lower Sand	17.3	F	1984 ± 73	IRSL_225_	13	0	547 ± 34	—	24	276 ± 20
				F	1984 ± 73	IRSL_50_	23	0	418 ± 10	—	18	*211 ± 10*
				Q	1196 ± 57	OSL	22	1	129 ± 9^c^	47	30	108 ± 10
									209 ± 16^c^	53		*175 ± 17*
	Shfd15023	Meriden Red Sand	7.55	Q	992 ± 57	OSL	20	0	96.2 ± 8.5^c^	28	24	97 ± 10
									149 ± 7.8^c^	72		*150 ± 12*
	Shfd16017	Meriden Lower Sand	14.4	Q	1226 ± 74	OSL	15	1	80 ± 11^c^	17	53	65 ± 10
									131 ± 18^c^	23		107 ± 16
									235 ± 15^c^	46		*192 ± 17*
									426 ± 59	01-Mar		347 ± 45
Park Farm	Shfd15024	Park Farm Sand and Gravel	8.1	Q	891 ± 49	OSL	19	2	160 ± 5.9	—	27	*180 ± 12*
	Shfd15025	Park Farm Upper Sand	2.9	Q	1046 ± 56	OSL	28	0	106 ± 7.6^c^	31	33	102 ± 9
									189 ± 8.1^c^	69		181 ± 12
Glebe Farm	Shfd17172	Baginton Sand Member	0.84	F	2259 ± 78	IRSL_225_	21	0	460 ± 18	—	19	203 ± 11
				F	2259 ± 78	IRSL_50_	24	0	322 ± 4.0	—	11	*142 ± 5*
				Q	1485 ± 78	OSL	21	3	83 ± 9.2^c^	24	40	56 ± 7
									178 ± 10^c^	76		120 ± 9
	Shfd17173		1.9	F	2264 ± 82	IRSL_225_	21	0	324 ± 10	—	20	*143 ± 7*
				F	2264 ± 82	IRSL_50_	24	0	239 ± 6.4	—	17	106 ± 5
				Q	1491 ± 82	OSL	22	1	39 ± 3.5^c^	37	83	26 ± 2.8
									87 ± 17^c^	34		59 ± 12
Wolston	Shfd17174	Wolston Sand and Gravel	1.25	F	2693 ± 106	IRSL_225_	24	0	415 ± 9.4	—	13	*154 ± 7*
				F		IRSL_50_	24	0	399 ± 3.9	—	7	*148 ± 6*
				Q	1920 ± 106	OSL	24	0	131 ± 13^c^	45	32	68 ± 8
									216 ± 18^c^	55		113 ± 11
	Shfd17175		1.58	F	2769 ± 111	IRSL_225_	21	0	418 ± 17	—	23	*151 ± 9*
				F	2769 ± 111	IRSL_50_	22	0	410 ± 5.9	—	9	*148 ± 6*
				Q	2005 ± 112	OSL	22	2	170 ± 18^c^	55	46	85 ± 10
									275 ± 42^c^	36		*137 ± 22*
Seisdon	Shfd15020	Siesdon Sand and Gravel	16	F	2531 ± 108	IRSL_225_	21	0	511 ± 19	—	19	*202 ± 11*
				F	2531 ± 108	IRSL_50_	24	0	701 ± 20	—	20	277 ± 14
				Q	1747 ± 106	OSL	21	0	219 ± 12^c^	46	89	125 ± 10
									413 ± 30^c^	27		*236 ± 22*
	Shfd15021		11.85	Q	1453 ± 81	OSL	23	0	163 ± 12^c^	27	68	112 ± 10
									304 ± 56^c^	56		*209 ± 15*

^a^Measurement type. OSL@50°C on quartz, IRSL@50°C on feldspar, IRSL@225°C on feldspar after IRSL measurement at 50°C.

^b^Number of aliquots measured that met the recycling criteria of 1 ± 0.1 and which were not saturated.

^c^Reported *D*_e_ based on Finite Mixture Modelling.

^d^Proportion of *D*_e_ replicates measured falling within a given Finite Mixture model extracted *D*_e_ component.

^e^Calculated ages with those shown in italics accepted on the basis of stratigraphy, sedimentology and luminescence data.

Some palaeogeographical deliberation has been given to the concept of the ‘*Bytham*’ River, particularly whether its alignment across the English Midlands and East Anglia could be sustained across a varied topography. Gibbard *et al.* [53] considered that the correlation of lithostratigraphical units has ignored the wider palaeogeographical evidence for two or more major lowland glaciations and the complex fluvial reorganization during the Quaternary. The correlation of Anglian Stage deposits in East Anglia with the Wolston Formation sediments in the English West Midlands is solely based on stratigraphic research in East Anglia. Moreover, the sites at Quinton and Nechells, near Birmingham, which preserve temperate interglacial lake deposits correlated to the Hoxnian Stage, both overlie and underlie glacial deposits that represent evidence of glaciation across the region during both the Anglian (Nurseries Till Formation) and Wolstonian (Ridgeacre Till Formation) stages [15,21]. It is also important to emphasize here that the Hoxnian Interglacial sediments reported at the Gilson locality, near Coventry underlie Wolston Formation-equivalent sediments [7,67,68], thus providing an additional fixed point in the apparent lack of independent stratigraphical control within the sequence.

The ancient Thames headwaters traversed the English West Midlands, from the present-day Upper Severn Valley [69], flowing through Evesham and into the today's River Evenlode [53] (figure 1). The Anglian Glaciation brought a significant re-organization of the river systems, sufficient to divert the ancient Thames’ headwaters into the Severn and the proto-Soar rivers [53,70]. Belshaw *et al.* [70] concluded that the major preglacial drainage system in southern England was dominated by consequent streams flowing from northwest to southeast, adjusted to the regional geological dip. This system was largely destroyed by glaciation during the Anglian Stage. A new drainage system later developed on deglaciated terrains as subsequent streams flowing along geological strike (i.e. northeast–southwest) eroded frost-susceptible clay bedrock under periglacial and permafrost conditions [71], beheading the courses of some older consequent streams. The proto-Soar River (figure 3) was one such post-Anglian subsequent stream, whose course was later destroyed by renewed glaciation during the Wolstonian Stage.

The aim of the present study is to elucidate the glacial dynamics, timing and interaction of late Middle Pleistocene events that deposited glaciogenic sediments across the English West Midlands district. The objectives are: (i) to establish a chronology for glacial and deglacial events based on optically stimulated luminescence (OSL), infrared stimulated luminescence (IRSL) and ^36^Cl exposure ages in combination with a reassessment of the Quaternary stratigraphy of the region; (ii) to reconstruct the palaeoenvironmental conditions of the region, particularly the timing of the glacial dynamics; and (iii) to correlate the late Middle Pleistocene history of the region with that of the wider glacial history of Britain and continental Europe. First, the regional setting of the English West Midlands is summarized.

## 2. Regional setting

### 2.1. Physiography and bedrock geology

The English West Midlands comprises two main physiographic units: the dissected plateau near the city of Birmingham (incorporating the Upper Severn and Tame valleys) and the expansive low-relief valley of the ‘Warwickshire Avon’ river to the southeast (figure 1). The area is underlain principally by the Triassic Mercia Mudstone Group, which is widely overlain by glaciogenic sediments. The city of Birmingham is characterized by a plateau divided by the river valleys of the Upper Severn and Tame. In the west, the Upper Severn Valley is incised, north to south, into mainly Devonian bedrock. The land surface rises to the Birmingham plateau formed mainly by Triassic Bromsgrove Sandstone, with the highest ground around Quinton (224 m above Ordnance Datum (OD)) and Frankley (250 m OD) [72,73]. The Tame Valley, west to east, is joined by its tributary, the River Blythe around Coleshill, turning north and formed on Mercia Mudstone. The Warwickshire Avon area hosts glacial sediments that infill some of the eastern region and overlie relatively soft Triassic Mercia Mudstone. Higher ground around Coventry is formed by Carboniferous Lower and Middle Coal Measures. The River Avon has incised a wide valley that runs northeast to southwest. Farther southeast lies the Jurassic escarpment, which rises to more than 200–250 m OD [11].

### 2.2. Pleistocene geology

**Figure 4.**
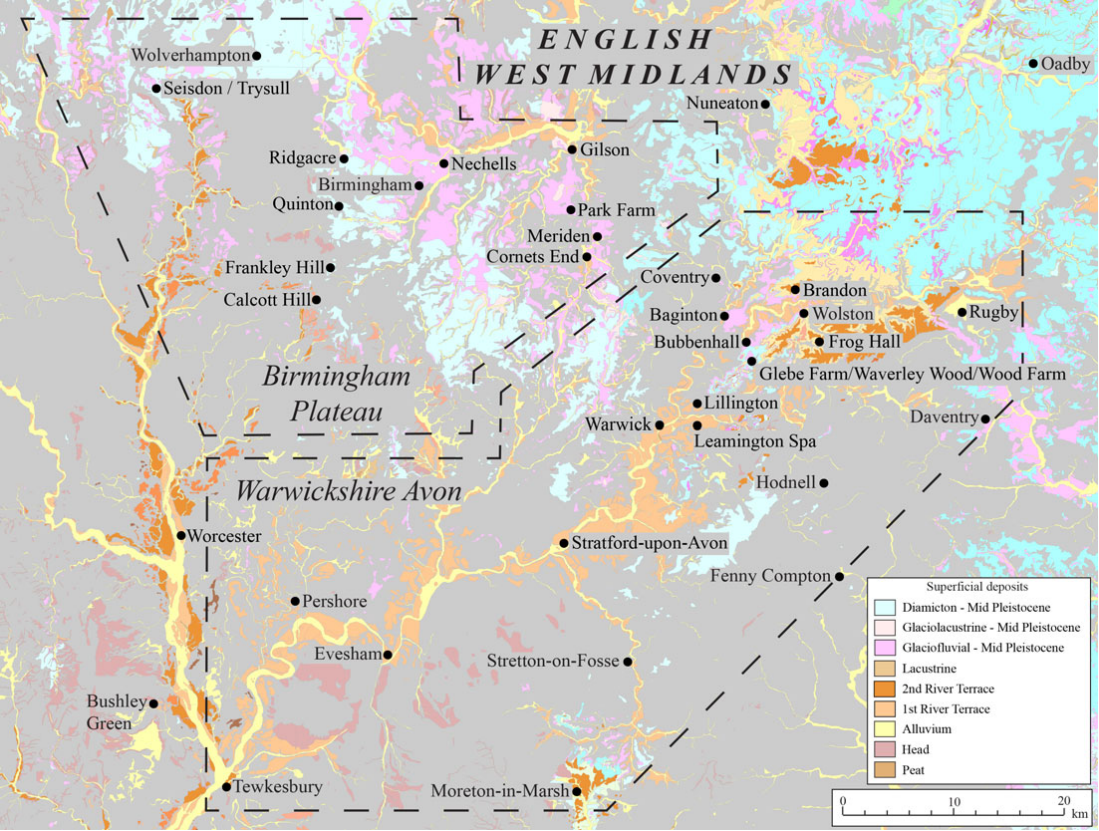
Map of the superficial deposits of the Birmingham plateau and Warwickshire Avon areas of the English West Midlands. The dashed line marks the boundary of the Birmingham and Warwickshire Avon areas discussed here. Modified from Digimap [74]. Light grey indicates an absence of superficial deposits.

The distribution of Pleistocene deposits previously mapped [11,49,72,73] in the English West Midlands is shown in figure 4 and descriptions and interpretations of them are summarized in table 1. Glaciation during the Anglian Stage in the region is sparsely understood and evidenced by two glacial diamictons. One is at Quinton, in the Birmingham plateau, where the Nurseries Till Member was deposited within a valley incised by sub-glacial scouring or sub-glacial meltwater erosion before the Anglian Stage [21,73,75]. The second is at Bubbenhall, in the Warwickshire Avon, where the Bubbenhall Till is believed to represent the sole evidence of Anglian glacial diamicton in the Avon Valley [1] (figure 1), although the so-called till has subsequently been demonstrated to show characteristics of a periglacial head deposit [10]. Other anecdotal evidence of Anglian glaciation has been reported at Nechells and Grimstock Hill, where both glaciogenic deposits underlie interglacial beds considered to have been deposited during the Hoxnian Stage [7,15,68]. No evidence has been reported that Anglian ice advanced further south into Warwickshire.

The majority of the cold-climate sediments within the English West Midlands have been considered by multiple authors to be of Wolstonian Stage (i.e. late Middle Pleistocene) age. Regionally, sediments predominantly comprise glacial (occasionally sandy) diamictons, glaciofluvial sand and gravels and glaciolacustrine silt, clay and sand. The most studied Wolstonian Stage cold-climate sediments occur around Wolston village, Warwickshire (figure 1).

Based on investigations by Shotton [1,3,18,26,36], Sumbler [10,78], Rice [60,61], Rice and Douglas [76], Douglas [77], the Wolston Glacigenic Formation [49] overlies a bedrock depression and represents the record of a cold-climate proto-river aligned southwest to northeast across the proto-Soar palaeovalley, from Evesham village, Warwickshire, to north of the city of Leicester (figure 1).

At the base of the sequence, the *Bagington-Lillington Gravel* Member (Baginton Formation: [49]) and overlying *Baginton Sand* Member (Baginton Formation: [49]) are mostly dominated by quartz-rich ‘*Bunter pebbles’* gravel and contain a reasonable selection of limestone, ironstone and flint. The gravel was deposited in a high-energy, cold-climate river system and the fine to medium, horizontally bedded sand represents the termination of the proto-Soar River, with the highest beds deposited in water impounded in front of an advancing glacier.

The *Thrussington Till/Moreton Till Member* (Wolston Glacigenic Formation: [6,13,49,60]) is predominantly a red to red–brown clay or sandy massive clay, its colour derived from Mercia Mudstone and Triassic rocks. Its clast content includes sandstone, siltstone, mudstone, quartz ‘*Bunter pebbles’* and coal, as well as rare Leicestershire diorites [60,61]. The till is generally 3–5 m thick across the region, but it does not occur above 90 m OD [11]. In the Tame Valley, the till contains rare erratic boulders (of rhyolite and andesite) derived from the Arenig volcanic rocks of North Wales.

With the first advance of glaciers, meltwater formed a glacial lake in which accumulated a series of intercalated beds of silt, clay and sand. The *Wolston Clay Member* of Shotton [1] (Wolston Glacigenic Formation: [49]) represents the main Pleistocene deposit of the English West Midlands. It consists of an upper and lower unit, separated by the *Wolston Sand and Gravel Member* (Wolston Glacigenic Formation: [49]). The clay is composed of either laminated clay or silt with lenses of quartz ‘*Bunter pebbles’* interpreted as dropstones [20]. Massive units are typically clay-rich and stoneless, representing deposition in a highly turbid lake [11,20]. The *Lower Wolston Clay Member* (Wolston Glacigenic Formation: [49]) overlies the *Thrussington Till Member/Moreton Till Member* (Wolston Glacigenic Formation [49]) around Leamington Spa–Brandon–Wolston (figure 1), where it has been reported to be up to 18 m thick at the village of Bubbenhall [11]. The *Lower Wolston Clay Member* thins to the east and south of Bubbenhall and is overlain by the *Wolston Sand and Gravel Member*. As identified by Shotton [1], the sand and gravel is typically 1–3 m thick over much of the eastern Warwickshire Avon area. They are thought to have been deposited as glaciofluvial outwash during a glacial advance across the glacial lake. The *Upper Wolston Clay Member* is the eastern lateral equivalent of the *Oadby Till Member* (see below) [10,61]. Old *et al.* [11] reported the maximum thickness of the upper unit as 50 m at Hillmorton. The stratigraphical association of the *Upper Wolston Clay* and the *Lower Wolston Clay* to overlying and underlying tills suggests the close proximity of glacial ice. The second ice advance into the region deposited the chalk-rich *Oadby Till Member* (Wolston Glacigenic Formation: [1,6,11,13,49,60,61]). It can generally be subdivided into upper and lower parts. The *Lower Oadby Till* contains mostly Late Triassic and Early Jurassic materials and quartzite pebbles [60,61]. The *Upper Oadby Till* is grey in colour and composed of Cretaceous and Middle Jurassic materials. Both the upper and lower parts of the till have a chalk-rich matrix [60,61]. In Warwickshire, it predominately contains weathered brown clay and clasts of chalk, flint, limestone and sandstone, with rarer occurrences of quartz ‘*Bunter pebbles’*, mudstone and coal. The *Moreton Till Member* at Stretton-on-Fosse is its equivalent, impounded against the Jurassic escarpment [60]. The Wolston Glacigenic Formation is completed by the *Dunsmore Gravel Member* [1,4,6,26,36]. It is a poorly sorted, sandy and clayey gravel on the plateaux of Dunsmore Heath and Knightslow Hill. Its lateral equivalent is within the *5th Terrace* of the Avon, mapped by Tomlinson [16,79,80].

### 2.3. Study sites

Five sites across the English West Midlands were selected for study, based on available exposures of Pleistocene sediments through quarrying or access to drilling (figures 1 and 4). Site 1 (British National Grid Reference: SP23524 82217) is at the Meriden Sand Pit, 0.2 km west of Meriden, in the Birmingham plateau. Site 2 (SP220785 83847) is at the Park Farm Pit, 3 km northwest of Meriden, in the Birmingham plateau. Site 3 (SP436852 272225) is at the Glebe Farm Pit, 0.4 km southeast of Bubbenhall in the Warwickshire Avon valley. Site 4 (SP41064 74620) is on the slope of the Dunsmore Heath ridge, 0.8 km south of Wolston Village in the Warwickshire Avon valley. Site 4 is the type site for the Wolstonian Stage in the British Isles [1,38] and is designated as the Wolston Site of Special Scientific Interest (SSSI). Site 5 (SO 84555 95080) is 1 km west of Seisdon, on the Birmingham plateau.

## 3. Methods

### 3.1. Stratigraphy and sedimentology

The stratigraphy and sedimentology at the five study sites were examined by logging of exposures and boreholes. Vertical sections were cleaned, and lithofacies and sedimentary structures were recorded and photographed, as per Evans & Benn [81]. Clast macrofabrics were measured from diamictons using samples of 50 clasts and processed in Geo-orient stereonet software. Palaeocurrent dips and directions were determined by a compass clinometer from sections exposing cross-bedded fluvial sediment. Boreholes were drilled where exposures of sediments were unavailable. Hand-augering with a 50 cm Dutch auger provided a quick and useful method to survey the sub-surface. A MRZB drilling rig was used to drill a borehole (5 m) at Wolston, where a combination of coring in difficult ground conditions and collection of sampled material for laboratory analysis was needed.

### 3.2. Geochronology

#### 3.2.1. Luminescence dating

**Figure 5.**
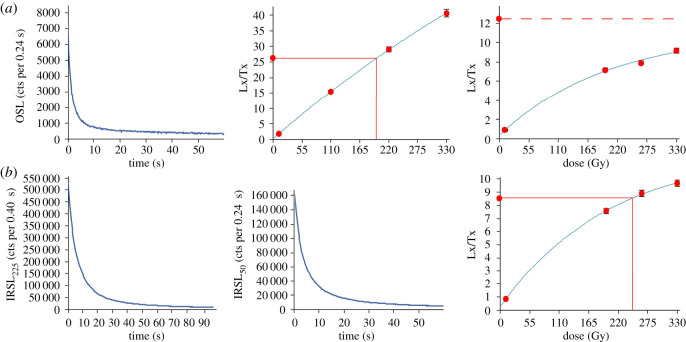
Examples of luminescence measurement data. (*a*) Quartz OSL shine down curve (left), SAR growth curve showing good growth with dose (middle) and SAR growth curve of aliquot where the signal has reached saturation (right). (*b*) Feldspar IRSL@225°C shine down curve (left), feldspar IRSL@50°C shine down curve (middle) and SAR growth curve for IRSL@225°C showing good growth with dose (right).

Eleven luminescence samples were collected from the five sites to establish when glacial, glaciofluvial and fluvial sands were deposited in the English West Midlands. Applying luminescence dating to date Middle Pleistocene sediments is challenging. Commonly, average background dose-rates lead to the saturation limit of quartz optically stimulated luminescence (OSL) being exceeded by around 150 000 years of burial or younger (e.g. [82]; figure 5*a*). The higher saturation dose limit of the feldspar infrared stimulated luminescence (IRSL) signal potentially extends the upper dating limit to 200 000–300 000 years or more, but when measured at 50°C it can suffer from anomalous fading and age underestimations [83]. However, it has been shown that fading can be reduced by making a second IRSL measurement at elevated temperatures (e.g. [84]). While high-temperature IRSL measurements potentially solve fading and saturation issues, unfortunately resetting of the IRSL signal is slower than for quartz OSL [85]. For glacial and fluvial sediments, in which the potential for sunlight exposure prior to burial may have been limited, this could lead to age overestimation (e.g. [46,86]). This study undertook both OSL of quartz and IRSL of feldspars (both at 50°C and 225°C referred herein as IRSL_50_ and IRSL_225_) in order to best understand the age of the sampled sediments.

Samples were prepared as per Bateman and Catt [87] using 7% HCl and 30% H_2_O_2_ to remove carbonates and organic materials and dry sieving to isolate grains 180–250 µm in diameter. Heavy minerals were separated off using lithium sodium tungstate (LST) at 2.7 g cm^−3^. Feldspars and quartz were separated using LST at 2.565 g cm^−3^. Quartz and feldspars were then etched in HF (48% HF for 40 min for quartz; 10% HF etch for 10 min for feldspar. All measurements were made as small aliquots (2 mm diameter samples mounted as a monolayer on 9.6 mm discs) with measurement made with a Risø TL-DA-15 automated luminescence reader.

Dose rates for the luminescence ages were determined from *in situ* field measurements made with an EG&G micronomad gamma-spectrometer or from laboratory inductively coupled plasma mass spectrometry (ICP-MS) converted to dose rates using data from Guérin *et al.* [88] (table 2). For feldspars an additional internal beta dose rate was derived using an assumed concentration of K of 12% [89] and Rb of 400 ppm [90]. The cosmic radiation contribution to the dose rate was calculated according to position and burial depth [91]. Total dose rates were attenuated by a near-saturation palaeomoisture value of 23 ± 5% based on sedimentary interpretations and accounting for quarry practices (most sites were being actively pumped dry).

Quartz OSL measurements were stimulated with blue–green diodes (emitting at approximately 470 nm) and the luminescence signal was detected through a Hoya U340 filter. OSL measurements were for 80 s at 125°C (figure 5*a*). OSL response to dose showed all samples were fairly insensitive to dose (dim). For example, Shfd17172 gave an initial OSL signal of 184 ± 26 OSL counts per unit dose, much lower than a bright Australian quartz for which values greater than 12 000 counts per unit dose are typical. The palaeodose (*D*_e_) was derived using the SAR protocol [92] (figure 5*a*). As feldspar contamination was detected in the prepared quartz by IRSL measurements, each OSL measurement within the SAR protocol was preceded by a 40 s IR wash at 50°C. A dose-recovery pre-heat plateau test [93] was used to optimize the pre-heat in the SAR protocol. Based on this a pre-heat of 160°C for 10 s was applied to samples Shfd15020-21 and Shfd17172-175 and of 180°C for 10 s to samples Shfd15022-25. Dose recovery at these preheat temperatures was within 1% of unity (*n* = 3). While measured quartz OSL *D*_e_ values for some samples were large (max 300 Gy), seven samples yielded a few (up to 12%) saturated aliquots (*D*_e_ more than twice *D*_0_ component of the exponential growth curve; [94]; table 2). However, most aliquots showed continued OSL signal growth with increased laboratory dose (figure 5*a*). Only aliquots with recycling ratios falling between 0.9 and 1.1 were accepted for further analysis.

Feldspar IRSL measurements were stimulated with IR diodes emitting at 870 nm and signal was detected through Schott BG-39 and CN 7-59 filters (figure 5*b*). *D*_e_ measurements were derived using the SAR protocol. IRSL measurements were made after a preheat of 250°C for 10 s with IRSL at 50°C (IRSL_50_) for 60 s followed by IRSL 225°C (IRSL_225_) for 100 s [95]. Measurement at 225°C was adopted rather than at higher temperatures as it reduces unwarranted sensitivity and test dose dependency issues reported elsewhere (e.g. [96,97]). At the end of each SAR cycle a thermal wash at 290°C for 100 s was applied to clean out traps prior to the next measurements. Fading measurements for IRSL_50_ were not conducted; instead *D*_e_ values were fading corrected with an assumed *g*-value of 2.5%/decade (along the lines of the Type iv approach of Rhodes [95]). While fading rates may have differed between samples and sites, differential fading rates were not considered appropriate without more knowledge on feldspar provenance for the different sedimentary units across the studied sites. Instead, IRSL_50_ age data were evaluated in comparison with the other methods employed, enabling identification and rejection of lower ages which had incompletely corrected fading.

Results of a sample exposed to UK sunlight for 7 days show the IRSL_225_ signal had a residual of 6.93 ± 0.11 Gy (*n* = 7), which was subtracted from all calculated final IRSL_225_ *D*_e_ values. Only aliquots with recycling ratios falling between 0.9 and 1.1 were accepted for further analysis.

**Figure 6.**
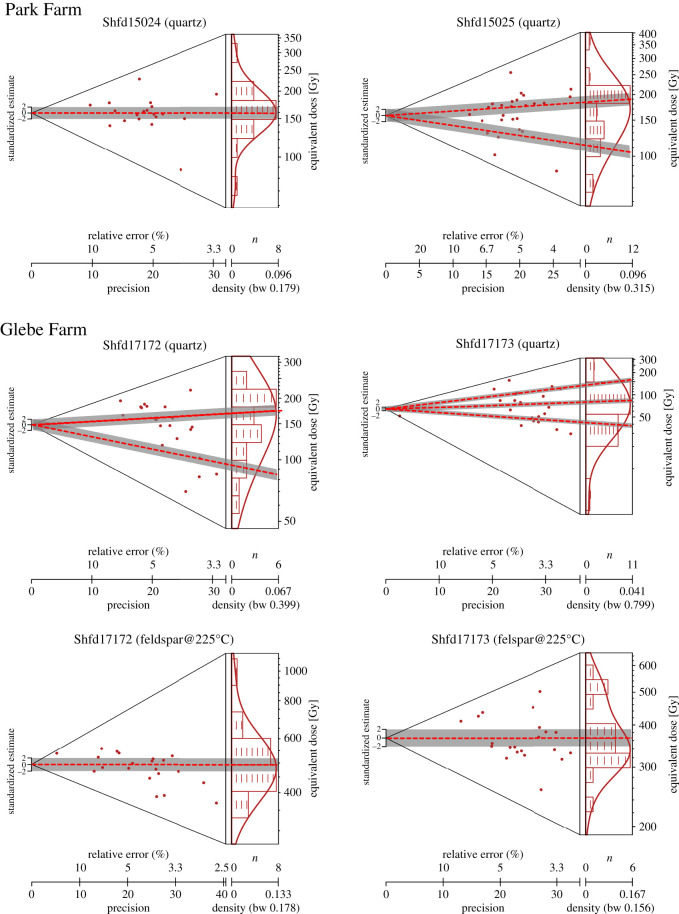
Example Abianco plots from Park Farm and Glebe Farm showing replicate equivalent dose (*D*_e_) scatter for OSL measurement of quartz and pIRSL_225_ measurements of feldspars.

A minimum of 24 replicates of each sample was measured to indicate the *D*_e_ reproducibility (figure 6). Samples with a unimodal *D*_e_ distribution were considered well bleached (reset) and the Central Age Model (CAM) used to provide an estimate of the mean *D*_e_ value and uncertainty for age calculation purposes [98,99]. For samples where the *D*_e_ replicate distribution was either multimodal or skewed, samples were considered incompletely bleached and in these instances a *D*_e_ value was calculated using the Finite Mixing Model (FMM; [98]). The *D*_e_ distributions of very small aliquots of British Isles glacial sediment with highly variable grain sensitivities have been shown to closely resemble the *D*_e_ distribution measured at the single grain level [100] (figure 5) and FMM has the advantage over minimum age models of allowing the discrimination of post-depositional disturbed sediment from those with the true burial dose [101].

#### 3.2.2. Exposure dating

Three samples for exposure dating were collected from erratics in the Birmingham area (figure 1, electronic supplementary material, figure S1). The likely source of the boulders is the Arenig volcanic rocks in the Arenig Mountains in northern Wales. The boulders are associated with the Frankley Hill Diamicton [68], itself part of the Ridgeacre Till Member [5]. Because of the absence of separable quartz, samples were processed for whole rock ^36^Cl (see electronic supplementary material, S1). All boulders were collected in hedgerows or on field boundaries. Because the area south of Birmingham city has been farmed for centuries, we cannot rule out the possibility that the boulders have been moved to these locations. The exposure ages for an additional three samples which demonstrated ‘glacial features’ from Phillips *et al.* [102] were re-calculated using the same procedures for comparison. These samples were taken from boulders around Calcott Hill [68,102] (figure 1) and are associated with the Ridgacre Till of Maddy [5] (see electronic supplementary material, S1 for further information).

## 4. Results

### 4.1. Stratigraphy and sedimentology

#### 4.1.1. Meriden

##### 4.1.1.1. Description

**Figure 7.**
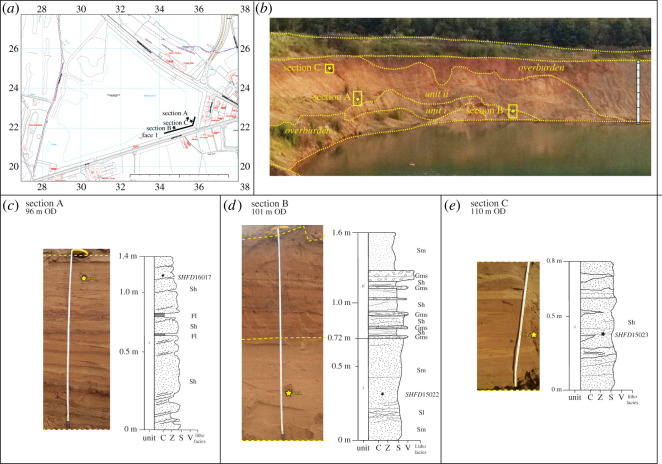
Pleistocene deposits at Meriden. (*a*) Location map, adapted from Digimap [74]. (*b*) Photograph of stratigraphy and location of sections A, B and C. (*c*) to (*e*) Sedimentological logs of sections A, B and C, respectively. Stars indicate location of luminescence dating samples.

Three sections (A–C) were logged along face 1 in the southeast corner of the Meriden Sand Pit (figure 7*a*). Face 1 provides a 22 m high exposure (figure 7*b*) that contains two lithostratigraphic units partially covered by overburden from quarry workings, as shown in figure 7*c*–*e* and detailed in table 3. In summary, unit i, a sand that is generally massive (Sm), is exposed centrally in the face, between 0 and 12 m marks of the face, with a maximum thickness of 5 m. Localized cross-bedding in the unit provided a palaeocurrent measurement indicating southward flow. Unit ii, a horizontal sand (Sh) with silty clay bedding (Fl), is consistent across the face, dipping east to west from 0 to 22 m marks of the face, with a maximum thickness of 17.5 m. The upper 2 m of the unit contain quartz and quartzite ‘*Bunter pebbles’*, as well as involutions.

**Table 3.** Lithostratrigraphy and sedimentology of face 1, Meriden. Oldest unit at base.

unit number	lithostratigraphical unit and thickness (m)	description	interpretation
ii	Meriden Red Sand (1.4–17.5)	Horizontal, planar and thickly laminated to thinly bedded medium red silty sand (Sh), with horizontal laminae to very thin beds of silty clay (Fl), matrix-supported massive gravel (Gms), and medium sand to silty sand; quartz ‘*Bunter pebbles’* dominate clast lithology; involutions in upper 2 m	Glaciolacustrine
i	Meriden Lower Sand (≤5)	Massive coarse sand (Sm), with occasional horizontal thin lamination of fine sand (Sh); local cross-bedding with southward palaeocurrent direction; gradational upper contact	Fluvial (moderate-energy) or possibly glaciofluvial

##### 4.1.1.2. Interpretation

The sedimentary sequence at Meriden is interpreted to record a change in depositional environment from fluvial to glaciolacustrine (table 3). The Meriden Lower Sand (unit i)—mainly coarse, massive sand with occasional thinly laminated fine sands—is attributed to deposition in a moderate-energy fluvial environment dominated by bedload transport (e.g. [103–105]) with a palaeocurrent direction towards the south. It probably represents a moderate-energy fluvial sand, infilling the Blythe Valley in a southwest to northeast direction, though a glacial meltwater source cannot be ruled out. It is consistent with a coarse sand, laid down by meltwater from the same northerly source of ice in the nearby Cornets End Quarry [7,36,73,79]. The overlying Meriden Red Sand probably accumulated within a glacial lake. Prominent horizontal strata within the sand are attributed to cyclic or rhythmic deposition, typical of a lacustrine or seasonally variable environment (e.g. [105]). The matrix-supported massive medium-bedded gravel within the sand suggests a ‘flashy’ regime water source proximal to the sequence, typical of glacial meltwater (e.g. [106]). The Meriden Red Sand is equivalent to a laminated fine sand reported by Shotton *et al.* [36] and Brown [7] in the nearby Cornets End Quarry, laid down within a proglacial lake. Observed periglacial/cryoturbated structures, within the top 2 m of the sedimentary sequence at Cornets End, post-date their host deposits and have been attributed to an extremely cold, dry periglacial environment during the Devensian Stage [7].

#### 4.1.2. Park Farm

##### 4.1.2.1. Description

**Figure 8.**
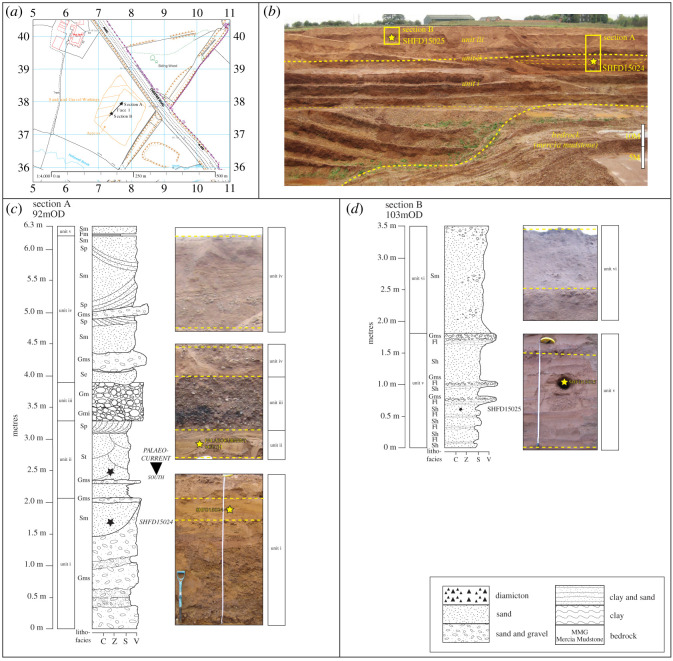
Pleistocene deposits at Park Farm. (*a*) Location map, adapted from Digimap [74]. (*b*) Photograph of stratigraphy and location of sections A and B. (*c*) and (*d*) Sedimentological logs of sections A and B, respectively. Stars indicate location of luminescence dating samples.

The Park Farm Pit is the location of the proposed ‘High Speed Two’ ‘Birmingham International Interchange’ railway station. Two sections (A and B) were logged along Face 1 in the northwest cutting of a sand pit at Park Farm (figure 8*a*). The face exposes a 13 m deep infilled channel cut into Mercia Mudstone bedrock (figure 8*b*). The Quaternary succession, which includes the infilled channel in the basal unit (i), can be divided into six lithostratigraphical units (figure 8*c*,*d*; table 4). In summary, units i to iv are dominantly sand and gravel, with planar or trough cross-bedding common, imbrication, massive gravel and southward palaeocurrents. Unit v is generally finer grained stratified sand and silty sand, with a prominent horizontal parallel stratification. Capping the sequence, unit vi is a massive fine to medium sand that grades into matrix-supported gravel table 5.

**Table 4.** Lithostratrigraphy and sedimentology of face 1, Park Farm. Oldest unit at base.

unit number	lithostratigraphical unit and thickness (m)	description	interpretation
vi	Park Farm Disturbed Sand (1.7)	Massive, fine to medium sand (Sm) grading into matrix-supported gravel (Gms); involutions in gravel dip down hillslope	Glacial lake deposit later disturbed by solifluction
v	Park Farm Upper Sand (1.8)	Cross-bedded (Fl) laminated silty sand (Sh) massive clay (Fm) bed; massive fine-to-medium sand (Sm) fine to medium horizontal planar very thinly bedded sand (Sh) with sharp contacts to fine, laminated, and silty clay (Fl) within the unit at 0.3, 0.45, 0.5, 0.7, 0.98, and 1.98 m. The unit grades upwards between the silty clay (Fl) and localized matrix-supported, horizontally bedded gravel (Gms). The contact with the overlying unit is gradational	Proximal glacial lake
iv	Park Farm Intermediate Sand (2.3)	Massive, medium to fine sand (Sm), with internal gradational contacts between planar cross-bedded medium sand (Sp) and lenses of matrix-supported gravels, with medium to fine sand (Gms); sharp upper contact	Glaciofluvial, with glacier nearby in Blythe valley
iii	Park Farm Gravel (0.5)	Discontinuous clast-supported, imbricated, gravel (Gmi) to north of face 1; imbrication dips towards south; increasing clast size to NW; erosional base; grades upward into clast-supported massive gravel (Gm); sharp upper contact	Outwash or subglacial meltwater channel deposit
ii	Park Farm Lower Sand (2)	Medium to coarse trough cross-bedded sand (St); top of unit marks transition into planar cross-bedding, with coarse to medium sand (Sp); southward palaeocurrent; sharp upper contact	Glaciofluvial, possibly glacial lake near top of unit
i	Park Farm Sand and Gravel (≤9.9 outside channel; 22.9 m within channel)	Matrix-supported, massive angular gravel and coarse to medium sand (Gms); irregular gradational contact within the unit to either a horizontal thinly bedded sand or an unsorted gravel (Gh/Sh) or massive sand (Sm) with a gradational upper contact infilled a channel feature	Proglacial braided river
Bedrock	Mercia Mudstone Group	Mudstone, very weak. Red, occasionally reddish brown mottled grey or black in pockets and lenses. Unbedded.

**Table 5.** Lithostratrigraphy and sedimentology of face 1, Glebe Farm. Oldest unit at base.

unit number	lithostratigraphic unit and thickness (m)	description	interpretation
iii	Glebe Farm (Thrussington) Diamicton (0.8)	Massive red–brown sandy diamicton; discontinuous; sub-rounded to rounded ‘*Bunter pebbles’* quartz, sandstone and limestone; many clasts striated	Subglacial traction till
ii	Glebe Farm (Baginton) Sand (0.2–3.35)	Stratified to massive medium to fine sand; sharp; contains quartz ‘*Bunter pebbles’*; trough cross-bedded to horizontally bedded sand; palaeocurrents mainly northeastward, with secondary directions towards north and east; sharp, undulating upper contact	Fluvial; lower energy environment than unit i
i	Glebe Farm (Baginton-Lillington) Gravel (1.2–3.4)	Matrix-supported gravel (Gms) containing rounded to sub-rounded coarse sand; clasts of quartz, quartzite ‘*Bunter pebbles’* and Mercia Mudstone; clast orientation is directed towards the north-northeast, and the clast-shape is compact–elongate; unit fines upwards with little gravel to the top of the unit; includes massive clay band (Fm) and massive, unsorted sand (Sm) with a gradational contact within the matrix; unit thins towards southwest; sharp to gradational upper contact	High-energy fluvial
Bedrock	Mercia Mudstone Group	Mudstone, very weak. Red, occasionally reddish brown mottled grey or black in pockets and lenses. Unbedded.	

##### 4.1.2.2. Interpretation

Overall, the sedimentary sequence at Park Farm is interpreted to record a change in depositional environment from a proglacial braided river to a glacial lake (table 4). The base of unit i has a scoured contact with the Mercia Mudstone Formation, which is typical of a fast-flowing river scouring bedrock [108,109]. The coarse, sandy to gravelly texture of units i to iv suggests relatively high-energy depositional conditions, and the trough cross-bedding, planar cross-bedding and imbrication are common in braided rivers [110]. The material infills the Blythe Valley in a southward direction and is equivalent to the glaciofluvial gravel reported by Cannell [14], which contains quartzite-rich, quartz and sandstone, mudstone, granite, rhyolite, andesite and coal of Welsh or Lake District provenance and infilling the Blythe Valley to the north [7,14]. Collectively, these three lower units are equivalent to the Wolston Sand and Gravel [5–7,26,36,60,61,73], which has been interpreted as outwash sand from the Thrussington glacier to the north of the region [6,61]. The massive gravel in unit i is suggestive of deposition as a longitudinal bar or as a gravel sheet [108]. The sandy and silty texture together with the horizontal planar parallel stratification suggest that the Park Farm Upper Sand (unit v) accumulated by rhythmic deposition as a proglacial lake deposit (cf. [106,111,112]), subject to episodic influxes of coarse sand and gravel from a nearby meltwater source (e.g. [113–115]). These sediments are equivalent to the Upper Wolston Clay of Shotton [1] and attributed to deposition within a glacial lake impounded by ice in the Severn catchment to the south and Oadby glacier to the north [6,61]. The Park Farm Disturbed Sand (unit vi) is interpreted as a glacial lake deposit that has been periglacially disturbed and soliflucted downslope. Periglacial disturbance is evidenced by involutions within the matrix-supported gravel. Similar observations were made in the nearby Cornets End Quarry by Brown [7].

#### 4.1.3. Glebe Farm

##### 4.1.3.1. Description

**Figure 9.**
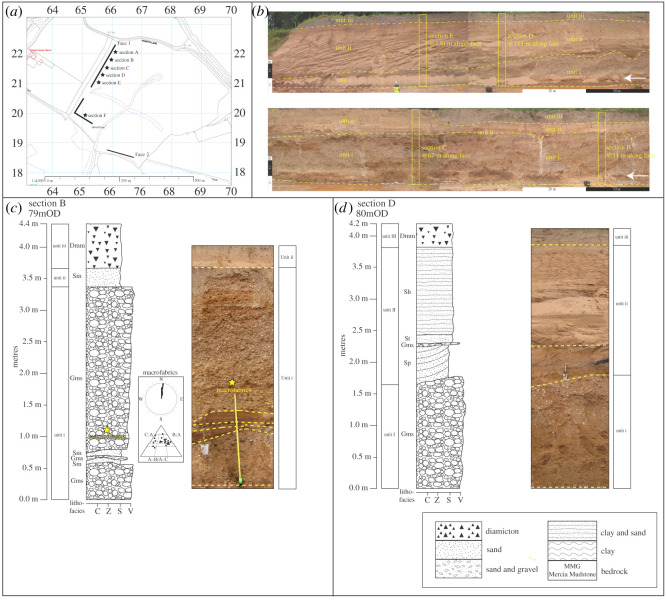
Pleistocene deposits at Glebe Farm. (*a*) Location map, adapted from Digimap [74]. (*b*) Photograph of stratigraphy and location of sections A to F. (*c*) and (*d*) Sedimentological logs of sections B and D, respectively. Stars indicate location of luminescence dating samples.

Gravel pit extraction has occurred around the Waverley Wood sites for several decades. The present excavation is that at Glebe Farm Pit (figure 9*a*). The sediments exposed here all form part of the Wolston Glacigenic Formation [1,10,11,20,26,36,49]. Six sections (A–F) were logged at Glebe Farm. Sections A–E were along face 1, and section F along face 2 of the working pit (figure 9*a*,*b*; table 5 electronic supplementary material, S2). Above Mercia Mudstone bedrock, the Quaternary sequence comprises three lithostratigraphic units. Unit i is a matrix-supported gravel with a coarse sand matrix. Clasts are predominantly quartz and quartzite ‘*Bunter pebbles’*. The sand matrix fines toward the top of the unit into fine to medium sand. The unit thins towards the southwest, with an average dip between 8° and 12°. Unit ii features a bedded medium to fine sand, within which the lowest 60 cm appear to be massive. The sand dips along the surface of unit i. After 60 cm the sand shows cross-bedding, though the uppermost part of the sand is horizontally bedded. Unit iii is a massive red–brown sandy diamicton, which is discontinuous across the face and has a sharp undulating contact with the underlying sand.

##### 4.1.3.2. Interpretation

The sedimentary sequence at Glebe Farm is attributed to deposition in a braided river system, shifting from higher to lower energy, before being overridden by glacial ice. Unit i (Baginton–Lillington Gravel) with its matrix-supported gravel and mainly sub-rounded to rounded clasts is typical of a bedload-dominated, high-energy fluvial system (e.g. [108,115–117]). It is equivalent to the Baginton–Lillington Gravel described by Shotton [1,3,6], Crofts [118] and Old *et al.* [11] at the Waverley Wood site. Unit ii (Baginton Sand), characterized by trough cross-bedded medium to fine sand, with occasional massive sand in pockets (sections B and C; figure 9*c*, electronic supplementary material, figure S2), is thought to have been deposited by water in a lower energy environment, within a series of channels, forming trough cross-bedded sand as transverse bars and dunes typical of fluvial deposition. Palaeocurrent directions (northeasterly, north and east) indicate flow was predominately to the northeast. It is equivalent to the Baginton Sand described by Shotton [1,3,6], Crofts [118] and Old *et al.* [11]. Unit iii (Thrussington [Till] Diamicton), which contains striated clasts, is interpreted as a subglacial traction till. The diamicton is equivalent to the Thrussington Till, discussed by Rice [60,61], Shotton [6], Crofts [118] and Old *et al.* [11]. Within it, erratics of Leicestershire diorites, Coal Measures ironstones and Lower Carboniferous limestone indicate that the glacier moved across the region from the northwest.

#### 4.1.4. Wolston

##### 4.1.4.1. Description

**Figure 10.**
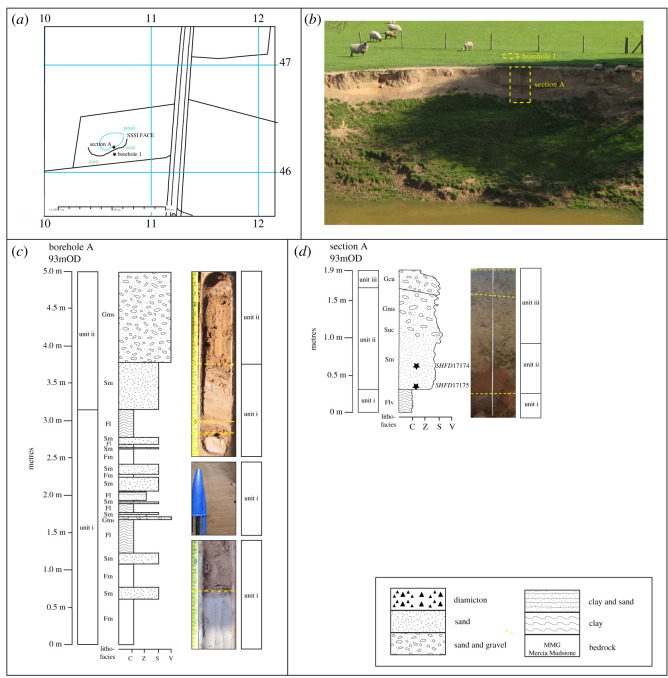
Pleistocene deposits at Wolston. (*a*) Location map, adapted from Digimap [74]. (*b*) and (*c*) Photographs and sedimentological log of borehole 1 and section A, respectively.

The Wolston site forms the stratotype of the Wolstonian Stage in the British Isles [1,38]. Within the Wolston SSSI site boundary, one borehole (Wolston SSSI borehole 1; SP41046 74620) and one section (Wolston SSSI section A; SP41064 74625) were logged along a slumped face identified in Old *et al*. [11] and Shotton [119] (figure 10*a*). Three lithostratigraphical units are identified using terms previously set out by Shotton [1,36,119], Sumbler [10], Old *et al.* [11] and Lewis [20] (table 6). Unit i (the Lower Wolston Clay) consists of laminated to massive clay with sandy to gravelly interbeds. Unit ii (the Wolston Sand and Gravel) is massive sand and matrix-supported gravel, grading upwards into unit iii (Dunsmore Gravel), a largely matrix-supported gravel.

**Table 6.** Lithostratrigraphy and sedimentology of Wolston. Oldest unit at base.

unit number	lithostratigraphic unit and thickness (m)	description	interpretation
iii	Dunsmore Gravel (0.3)	Matrix-supported gravel containing massive medium sand (Gcu); sub-angular to sub-rounded clasts	Glaciofluvial sand and gravel, with or without some periglacial reworking (e.g. by solifluction)
ii	Wolston Sand and Gravel (1.3)	Massive medium sand (Sm) gradually coarsening upwards (Suc) into a massive matrix-supported gravel (Gms) with angular to sub-angular clasts; gradational upper contact	Glaciofluvial outwash
i	Lower Wolston Clay (>3)	Thinly laminated grey–brown clay (Fl) or massive clay (Fm), with interbeds of massive medium to coarse sand (Sm), laminated silt, or matrix-supported angular to sub-angular gravel; sharp upper contact	Proglacial lake deposits, with increasingly proximal meltwater source

##### 4.1.4.2. Interpretation

The sedimentary sequence at Wolston is attributed to deposition within a proglacial lake that was subsequently buried by glaciofluvial outwash [1]. The laminated clay of unit i (Lower Wolston Clay) is typical of a lake deposit, and the interbeds of silt, sand or gravel suggest sediment sources of varying texture and/or fluctuations in energy conditions, all features common to glacial lakes [106]. The gravel bed at 3.35 m depth (figure 10*b*) may indicate a glacial meltwater source into the lake, and the overall coarsening-upward texture of unit i may record a meltwater source that became increasingly proximal through time (e.g. [20,107]). The likely source of meltwater was the glacier that deposited the Thrussington [Till] Diamicton in the local area (table 6). Similar observations were made by Douglas [120] around Market Bosworth. Furthermore, Lewis [20] reported small clasts in the Lower Wolston Clay resembling those from the Thrussington [Till] Diamicton (Bromsgrove (Keuper) sandstone, coal and erratics from Leicestershire and Nuneaton igneous rocks). The sand and gravel of units ii (Wolston Sand and Gravel) and iii (Dunsmore Gravel), which include sub-angular to angular clasts, are typical of glaciofluvial deposits [110], possibly subject to some periglacial reworking (e.g. by solifluction; [119]).

#### 4.1.5. Seisdon

##### 4.1.5.1. Description

**Figure 11.**
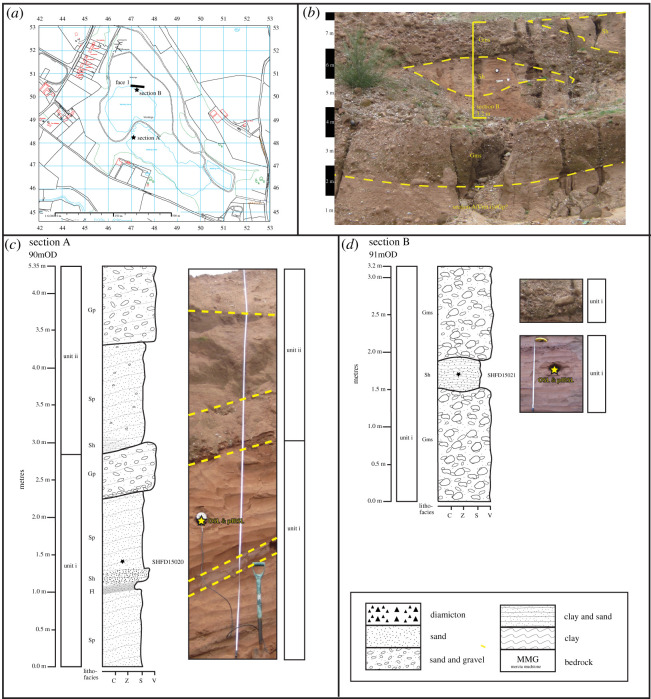
Pleistocene deposits at Seisdon. (*a*) Location map, adapted from Digimap [74]. (*b*) Photograph of stratigraphy and location of sections A and B. (*c*) and (*d*) Sedimentological logs of sections A and B, respectively. Stars indicate location of luminescence dating samples.

At Seisdon, two sections (A and B) and one face [1] in the current working pit were logged (figure 11*a*). Two lithostratigraphical units are identified in the present study, though due to complexity of the deposits at Seisdon, Morgan [17] found it impossible to correlate individual units in the pit. Unit i (Seisdon Sand) is a discontinuous, horizontal, thinly bedded, fine to medium sand containing some silty clay and coarse sand. The sediments dip between 22 and 25°. Unit ii (Seisdon Gravel) is a horizontally bedded, matrix-supported gravel with horizontal, thinly bedded fine to medium sand.

**Figure 12.**
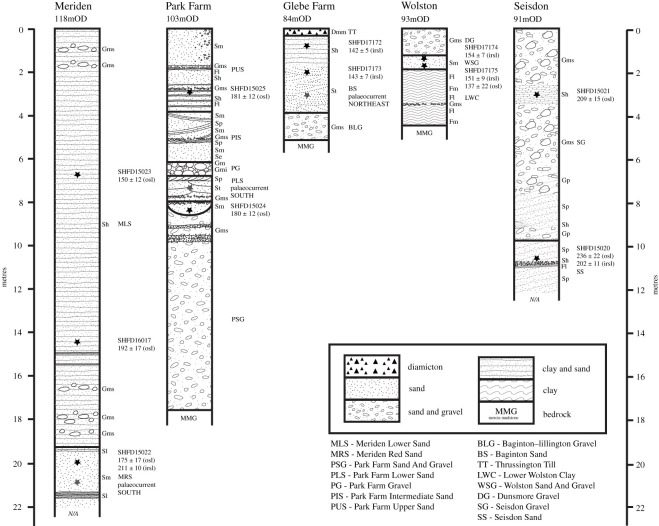
Composite multiple logs of the five sites (Meriden, Park Farm, Glebe Farm (Waverley Wood), Wolston and Seisdon) with luminescence ages and palaeocurrents indicated by stars. Elevations (m above Ordnance Datum, OD) refer to the top of the sections.

##### 4.1.5.2. Interpretation

Overall, both lithostratigraphical units at Seisdon are interpreted as fluvial or glaciofluvial in origin. The generally coarse-grained nature and stratification of the deposits, and the rounded to well-rounded clasts, are consistent with deposition in a relatively high-energy braided or anastomosing river system (e.g. [108]). The sandstone clasts of local Keuper Sandstone and predominantly ‘*Bunter pebbles*’ lithologies in the Seisdon Gravel imply a local river system in the valley, as the material is locally sourced, although it is not possible to exclude a glaciofluvial source. Deformation of the Seisdon Sand has resulted in some tilting of some strata, though the cause is not known.

### 4.2. Geochronology

#### 4.2.1. Luminescence dating

Applications of luminescence dating to glacial and glacial proximal sediments can be challenging, and so the results required careful analysis and interpretation. The latter used the luminescence replicate data, different characteristics of the luminescence signals measured (in terms of bleachability, saturation and fading) as well as sedimentological information (law of superposition, site stratigraphy, whether sediments were glacial/interglacial in origin).

##### 4.2.1.1. Meriden

At the Meriden site three samples were collected for luminescence dating: two from the Meriden Lower Sand (Shfd15022 and Shfd16017) and one from the Meriden Red Sand (Shfd15023). Quartz OSL *D*_e_ replicate distributions for all three were multimodal, which was taken to indicate only partial bleaching had occurred prior to burial. For partially bleached sediment, ages would normally be based on lowest *D*_e_ values (assumed to contain the best bleached grains and therefore closest to true burial dose). Such an approach when applied here using the lowest FMM *D*_e_ component produced ages which fell within the Ipswichian Interglacial Stage (*ca* MIS 5e; figure 12). Given the glacial depositional context of the sediments (described above) quartz age underestimation is suspected as reported elsewhere (e.g. [121]). This may be due to the dim nature of the OSL signal but also may be attributable to a hard-to-bleach feldspar contribution to the OSL signal not removed after the 40 s IR wash at 50°C within the SAR measurement. Accepting the dominant FMM *D*_e_ component yielded quartz OSL ages of 192 ± 17 ka (Shfd16017), 175 ± 17 ka (Shfd15022) and 150 ± 12 ka (Shfd15023; table 2). These ages increase in antiquity with depth and produce ages for Shfd16017 and Shfd15022, which were taken from the same unit, within errors of each other. Sample Shfd15022 also produced a feldspar IRSL_50_ age of 211 ± 10 ka and an IRSL_225_ age of 276 ± 20 ka (table 2). The much higher age for the IRSL_225_ measurement compared with both the IRSL_50_ and OSL ages suggests this sample had some sunlight exposure but insufficient to reset the harder to bleach IRSL_225_ signal, leading the IRSL_225_ age to be an overestimate. In summary, the sediments from the Meriden site were probably deposited between 150 ± 12 ka and 211 ± 10 ka, with ice-proximal deposition (Oadby Till) after 175 ± 17 ka.

##### 4.2.1.2. Park Farm

Two luminescence samples were collected from the Park Farm. Sample Shfd15024 from the outwash or a subglacial meltwater channel deposit of the Park Farm Sand and Gravel and Shfd15025 from the glaciolacustrine Park Farm Upper Sand. For Shfd15024 the OSL *D*_e_ replicates were unimodally distributed (figure 12), suggesting this sample was fully bleached at deposition. It returned an age of 180 ± 12 ka (table 2). Sample Shfd15025 had a multimodal *D*_e_ replicate distribution when measured by OSL (figure 12). FMM isolated two components. Just as with the Meriden samples, the lowest *D*_e_ component returned an implausibly young age within the Ipswichian Interglacial Stage (*ca* MIS 5e) and are assumed to have suffered from a dim contaminated OSL signal. As a result the age of 181 ± 12 ka from the larger FMM component was preferred. The overlapping of both ages is concordant with the sedimentological evidence for near-continuous deposition at this site. In summary, the age of the Park Farm Sand and Gravel and Park Farm Upper Sand appears to be 180 ± 12 ka.

##### 4.2.1.3. Glebe Farm

Sampling for luminescence targeted the cross-bedded and massive medium glacial sand of the Baginton Sand Member of the Wolston Glacigenic Formation. Two samples (Shfd17172 and Shfd17173) were collected and both had a multimodal *D*_e_ replicate distribution when measured by OSL (figure 12; table 2). FMM isolated two and three components in the dim OSL data respectively but all returned implausibly young ages of Ipswichian Stage Interglacial or younger so have been disregarded. The IRSL_50_ ages produced a young age for Shfd17173 which is seen as an underestimate and an age of 142 ± 5 ka for Shfd17172 (table 2). The IRSL_225_ measurements yielded ages of 207 ± 11 ka (Shfd17172) and 143 ± 7 ka (Shfd17173; figure 12; table 2) but the errors on these do not overlap and they are stratigraphically reversed. Given the coincidence of the IRSL_50_ for Shfd17172 and IRSL_225_ for Shfd17173, these ages have been tentatively accepted (assuming the older IRSL_225_ age for Shfd17172 is due to less bleaching). This gives a combined age (combined using OxCal v. 4.4.3; [122]) of 143 ± 4 ka (Late Wolstonian Substage, *ca* MIS 6a). However, given the uncertainty within the data it is suggested that this is used only as a minimum age for the deposition of the Baginton Sand Member and the Thrussington Till.

##### 4.2.1.4. Wolston

Luminescence sampling targeted the Wolston Sand and Gravel Member of the Wolston Glacigenic Formation with two samples collected. OSL measurement indicated the samples were dim and poorly bleached based on the scatter of *D*_e_ replicates. FMM isolated two components for both samples but just as with the Meriden samples the lowest component returned ages within the Ipswichian Stage Interglacial as did the older FMM *D*_e_ component for Shfd17174. Sedimentologically, deposition of the sand and gravel member during an interglacial was unlikely (e.g. [123]). The older FMM *D*_e_ components for Shfd17175 returned an age of 137 ± 22 ka but given the problematic nature of the OSL results could not be accepted without corroboration. IRSL_50_ ages for these samples produced ages of 148 ± 6 and 148 ± 6 ka. pIRSL_225_ measurements yielded ages of 154 ± 7 ka (Shfd17174; figure 12) and 151 ± 14 ka (Shfd17175; figure 12; table 2). The consistency of the ages from samples taken in close proximity from the same sedimentary Member and the concordance with a sedimentological non-interglacial time interval is taken to indicate these are true burial ages. The quartz OSL age for Shfd17175 also is within errors of these. Based on this the Wolston Sand and Gravel Member has a combined age (combined using OxCal v. 4.4.3; [122]) of 150 ± 3.3 ka placing it and the ice associated with these sediments to within the Late Wolstonian Substage.

##### 4.2.1.5. Seisdon

Two samples for luminescence dating were collected from the Seisdon Sand (Shfd15020) and Seisdon Gravel (Shfd15021). The glacial depositional context of the Seisdon Sand leads to a high probability of partial re-setting of the luminescence signal during deposition [124,125]. The site also underwent post-depositional deformation and tilting during subsequent late Devensian glaciation (*ca* MIS 2) [126]. FMM analysis of the dim quartz OSL results showed multiple *D*_e_ components for both samples, suggesting incomplete bleaching or disturbance. The lowest FMM *D*_e_ components for both samples yielded Ipswichian Stage Interglacial (*ca* MIS 5e) ages which are implausible based on the site sedimentology and its glacial nature (table 7; figure 12). Use of the larger FMM *D*_e_ component found in each sample produced ages of 209 ± 15 and 236 ± 22 ka. These agree well with the IRSL_225_ age for sample Shfd15020 of 202 ± 11 ka. Combining these ages (using OxCal v. 4.4.3; [122]), the best estimate of the age of the Seisdon sediments and associated proximal ice is 211 ± 7 ka (*ca* MIS 7b of the Late Wolstonian Substage).

**Table 7.** Lithostratrigraphy and sedimentology of Seisdon. Oldest unit at base.

unit number	lithostratigraphic unit and thickness (m)	description	interpretation
ii	Seisdon Gravel (3.05–3.2)	Matrix-supported gravel (Gms) with medium to coarse matrix and well-rounded clasts; clasts predominantly sandstone from local outcrops, with ‘*Bunter pebbles*’ quartzite also present; contains trough-shaped body of coarse sand and silty sand with horizontal bedding (Sh); horizontal, thinly bedded, fine to medium sand (Sp) and dipping between 22^o^ and 25^o^ with occasional rounded to well-rounded pebbles; matrix-supported, massive gravel, with mainly ‘*Bunter pebbles*’	Braided (fluvial or glaciofluvial) channel
i	Seisdon Sand (2.3)	Horizontal, thinly bedded, fine to medium sand (Sp), dipping between 22^o^ and 25^o^; includes laminated silty clay (Fl) with a gradational contact with underlying sand; clay bed dips at 25^o^	Fluvial or glaciofluvial

##### 4.2.1.6. Luminescence dating summary

**Figure 13.**
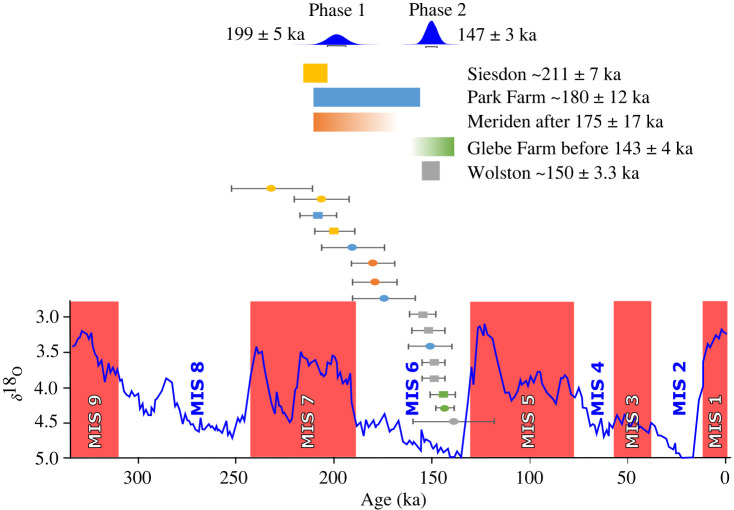
Accepted luminescence chronology for sampled sites from the English West Midlands placed into MIS with warm periods shown in red (data from Lisiecki and Raymo [28] ). Squares indicate feldspar ages, circles indicate quartz ages. Above are site summaries of when ice was proximal and ice phases derived from combining ages using OxCal.

Luminescence dating at Seisdon shows the glacier was proximal to the site at 211 ± 7 ka (*ca* MIS 7b) and proximal at the Park Farm site around 180 ± 12 ka (*ca* MIS 6e), within the Late Wolstonian Substage. The Meriden site indicates the deposition of the Oadby Till after 175 ± 17 ka (*ca* MIS 6e) and deposition of the Baginton Sand Member and the Thrussington Till at Glebe Farm before 143 ± 4 ka (*ca* MIS 6a). At Wolston the ice associated with the Wolston Sand and Gravel Member has an age of 150 ± 3.3 ka (*ca* MIS 6b). Analysis of the resultant age dataset in OxCal showed this dataset could not be combined into a single phase. FMM analysis of the ages with a sigma-b set at 0.07 to reflect the average uncertainty of individual ages identified two phases. OxCal defined these as an early phase at 199 ± 5 ka (*ca* MIS 7a) and a later one at 147 ± 2.5 ka during the Late Wolstonian Substage (*ca* MIS 6b; figure 13).

#### 4.2.2. Exposure dating

The new and revised exposure ages are presented in table 8, ages are presented with internal errors and external errors in brackets. The ages scatter more than expected and suggest that some of the boulders may not be *in situ*. All but one of the ages fall between 225 and 100 ka. The ages rule out deposition before the Wolstonian Stage and during the Early-to-Middle Wolstonian Substage (*ca* MIS 10–8). Weathering is unlikely to be the cause of the scatter, but periglacial action during the last glacial cycle could conceivably have heaved boulders. Erratics dated by Phillips *et al.* [102] were brought in by Wolstonian Stage ice but are only directly related to glacial diamicton at Calcott Hill (Sample GB-B1) (see electronic supplementary material, S1 for further discussion).

**Table 8.** ^36^Cl Exposure ages for the glacial erratics sampled in the English West Midlands. Ages are presented with internal errors and external errors in brackets.

sample	lab code	[^36^Cl]_n_ (10^5^ atoms g^−1^)	[^36^Cl]_c_ (10^5^ atoms g^−1^)	*P*_K_ %	*P*_Ca_ %	*P*_Cl_ %	exposure age (ka)
EGG-01	ANU-C303-21	0.066	9.440	83	0	16	225 ± 7(21)
FRH-01	ANU-C303-23	0.084	8.901	90	0	9	115 ± 3(9)
WAR-01	ANU-C303-24	0.066	4.189	90	0	9	52 ± 2(4)
GB-B1^1^		0.082	5.879	85	0	14	103 ± 13(15)
GB-B4A^1^		0.097	11.311	86	2	11	223 ± 18(26)
GB-B6^1^		0.102	10.212	86	1	12	155 ± 10(15)

^1^Philips *et al.* [102]

## 5. Discussion

### 5.1. Age of glaciation and drainage development in the Midlands

The distinct lack of widespread early Middle Pleistocene deposits in the region can be attributed to subsequent cold-climate periglacial erosion of the easily eroded Mercia Mudstone bedrock [68] and extensive glaciation during the Late Wolstonian Substage. Periglacial erosion rates during the Middle Pleistocene overlying Mesozoic argillaceous rocks such as the Mercia Mudstone varied between 0.20 and 0.42 m per 1000 years and suggest that the Jurassic escarpment was less developed in the landscape during the Anglian Stage than in the subsequent Wolstonian Stage [68,127–129] (figure 1). During the *ca* 250 000 years of the Wolstonian Stage, periglacial weathering and erosion of the Mercia Mudstone may have been of the order of 50–105 m [68]. This allowed the Jurassic escarpment to influence the limit of the approaching Late Wolstonian Substage ice because the escarpment now formed a significant topographical obstacle. The form of the Jurassic escarpment has implications for Middle Pleistocene glacial limits.

### 5.2. Late Wolstonian Substage

#### 5.2.1. Midlands

**Figure 14.**
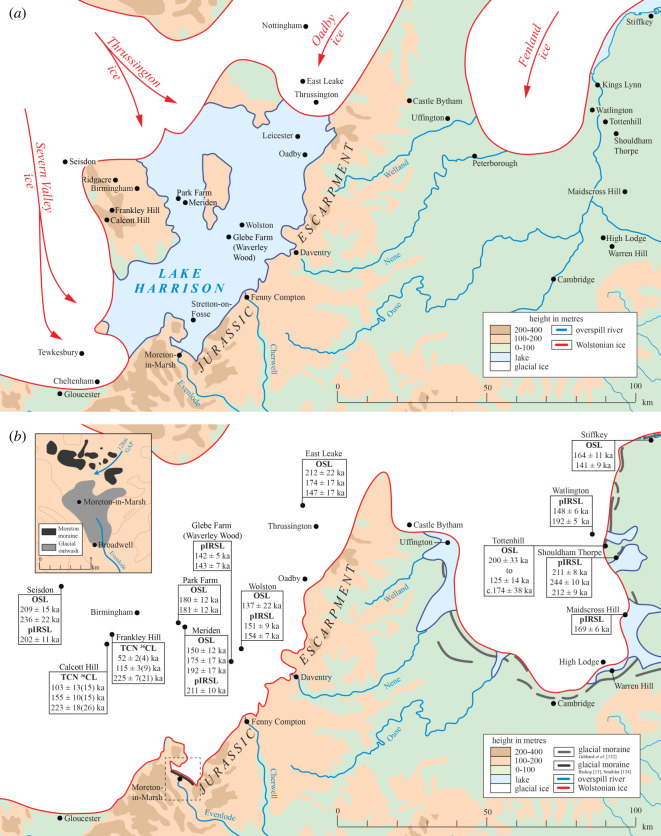
The Moreton Stadial palaeogeographical reconstruction/glacier dynamics during the Late Wolstonian Substage and its relative timing from luminescence and exposure dating. (*a*) Early Moreton Stadial glaciation and the maximum extent of glacial Lake Harrison. Arrows mark the ice-stream flow directions. Lake Harrison is modified from Shotton [1,26] and Bishop [13]. (*b*) The Wolstonian Stage glacial maximum during the Moreton Stadial in the English West Midlands. The glacial maximum is modified from: in the east [68,132,133] and in the west [1,6,13,26,36,67,131,134]. Inset shows the Moreton moraine, modified from Bishop [13] and Sumbler [134] (see appendix A). Note the glacial maximum is time-transgressive and the map represents the extent of ice during the entire Late Wolstonian Substage.

Glaciation during the Late Wolstonian Substage was the most important event in the Pleistocene history of the English West Midlands, based on the results presented herein (table 9). A revised Pleistocene stratigraphical nomenclature for the region is presented in table 10. Welsh ‘Arenig’ ice advanced into the west, initially from the Arenig Mountains of Snowdonia, in northwest Wales [1,9,102,131] (figures 1 and 14*a*). The Arenig ice infilled the upper Severn Valley (see above) and deposited glacial erratics across the region to the southwest of Birmingham plateau [8,9,102,131,135]. The exposure ages (table 8) allow us to discount both an earlier Anglian-age and that of the later Devensian glaciations, confirming a Late Wolstonian Substage glaciation of the English West Midlands.

**Table 9.** Sequence of major palaeoenvironmental events in the English West Midlands since the beginning of the Middle Pleistocene as proposed in the present study (modified from Shotton [1,6,34], Bishop [13], Rice [60], Old *et al.* [11], Bridge *et al.* [47], Maddy [5], Powell *et al.* [73], Gibson [8]).

stage	MIS	events			environment
		Avon Valley	Tame Valley	Severn Valley	
Holocene	1	Aggradation of alluvium and organic deposits on present river flood-plains			Interglacial
		1st river terrace deposits (Avon)	(Tame)		
Devensian	2	2nd river terrace deposits	(Hams Hall)	Advance of the Wolverhampton Till ice north-west of Birmingham/Wolverhampton	Glacial/Periglacial
		Downcutting of river valleys			
		3rd/4th river terrace deposits (Avon)			
	4	Periglacial reworking (solifluction)			
Ipswichian	5e	Aggradation of alluvium and rivers downcutting into underlying Wolstonian Stage deposits			Interglacial
Late Wolstonian Substage	6	Initiation of modern rivers (2) and downcutting			Fluvial
		5th river terrace deposits (Avon)			
		Periglacial re-working on post-glacial landscape (solifluction/thermal contraction cracking)			Periglacial
		Deposition of glacial outwash/initiation of modern rivers (1) (Dunsmore Gravel)			Glaciofluvial Glacial
		2nd period of Lake Harrison			
		Deposition of Upper Wolston Clay			
		Advance of Oadby Till ice across region	Deposition of Park Farm sands		
		Deposition of Wolston sands	Deposition of Meriden sands		
		1st period of Lake Harrison			
		Deposition of Lower Wolston Clay			
		Advance of Thrussington Till ice across region		Advance of Ridgacre Till ice across area	
Middle-Early Wolstonian Substage	7	Deposition of Baginton Sand in proto-Soar	Deposition of Park Farm sand and gravel	Deposition of Seisdon sand and gravel	Fluvial/Periglacial
	8 to 10	Deposition of Baginton gravels in proto-Soar	Aggradation of fluvial sand and gravel		
Hoxnian	11		Organic deposits at Gilson and Nechells	Organic deposits at Quinton	Interglacial
Anglian	12			Advance of Nurseries Till ice	Glacial
				Deposition of gravels, downcutting river valleys

**Table 10.** Proposed revised sequence of lithostratigraphical and chronostratigraphical events in the English West Midlands, revised from Shotton *et al.* [34] and Maddy [5]. *The Waverley Wood Sand and Silt Member has been correlated with the Early-to-Middle Wolstonian Stage, likely pre-glaciation during *ca* MIS 7-10 [130].

stage	MIS	event	Avon Valley			Tame Valley			Severn Valley (Birmingham)			
			*Members*	*Formation*	*Luminescence Dating*	*Members*	*Formation*	*Luminescence Dating*	*Members*	*Formation*	*Luminescence Dating*	*Exposure Dating*
Holocene	1		1st Avon Terrace	AVON VALLEY	* *	1st Tame Terrace		* *			* *	* *
Devensian	2		2nd Avon Terrace			Hams Hall Terrace			Wolverhampton Till	STOCKPORT		*52 ± 2(4) ka, 103 ± 13(15) ka, 115 ± 3(9) ka*
Ipswichian	5		3rd Avon Terrace						Ambercote Gravel			
			4th Avon Terrace									
Late Wolstonian Substage	6		5th Avon Terrace									
			Dunsmore Gravel	WOLSTON	*137 ± 22 ka, 142 ± 5 ka, 143 ± 7 ka, 151 ± 9 ka, 154 ± 7 ka*	Meriden Red Sand	MERIDEN	*150 ± 12 ka, 175 ± 17 ka, 180 ± 12 ka, 181 ± 12 ka, 192 ± 17 ka, 211 ± 10 ka*		RIDGEACRE	*202 ± 11 ka, 209 ± 15 ka, 236 ± 22 ka *	*155 ± 10(15) ka, 223 ± 18(26) ka, 225 ± 7(21) ka*
		Moreton	Upper Wolston Clay/Oadby Till			Meriden Lower Sand						
			Wolston Sand & Gravel			Park Farm Gravel						
			Lower Wolston Clay/Thrussington Till/Moreton Till			Park Farm Lower Sand						
						Park Farm Sand and Gravel			Ridgeacre Till/Frankley Hill Till			
			Baginton Sand	BAGINTON	* *							
			Baginton-Lillington Gravel									
Middle-Early Wolstonian Substage	7 to 10											
			Waverley Wood Sand and Silt	WAVERLEY WOOD	* *	Gilson Clayey Sand	GILSON	* *	Trysull Organic Bed	SEISDON	* *	* *
									Trysull Sand & Gravel			
Hoxnian	11				* *	Gilson Organic		* *	Quinton Silt & Peat/Nechells Organic	QUINTON	* *	* *
Anglian	12				* *	Gilson Sand and Silt		* *	Nurseries Till	NURSERIES	* *	* *
									Halesowen Beds			

The proglacial Lake Harrison developed in front of the advancing ice and the Jurassic escarpment. Lake water was impounded by the advancing Thrussington ice from the northwest, Oadby ice from the northeast, overriding the pre-existing proto-Soar valley, Severn Valley ice acting as a barrier to glacial meltwater to the west around Tewkesbury–Gloucester, and by the Jurassic escarpment to the south and southeast [1,6,11,13,60,61,79]. Lake deposition was interrupted by the two main phases of glaciation (i.e. the advance of the Thrussington ice and the advance of the Oadby ice) [6,13,16,134]. The major outlets of Lake Harrison included Daventry (which led into the proto-Nene palaeovalley) at 128 m OD, Fenny Compton (which led into the Cherwell (Upper Thames) Valley) at 129 m OD, and Moreton-in-Marsh (which led into the Evenlode (Upper Thames) Valley at 130 m OD (figures 1 and 14). The overflow through the Fenny Compton and Moreton-in-Marsh gaps, during the two stages of the ice advance into the lake, resulted in the aggradation of glaciofluvial gravel in the Cherwell and Evenlode valleys (figures 1 and 14) as the Wolvercote Terrace deposits, which contain glacial erratics that correlate unequivocally to the Oadby Till ice [13,16,26,134]. The Wolvercote Terrace sequence of the Upper Thames provides a critical independent stratigraphical control on the timing of glaciation, since it is interpreted as representing the equivalent to the Taplow Terrace Gravel Member in the Middle to Lower Thames, which is of Late Wolstonian Substage age [5,13,26,136].

Following glacial deposition, the lake re-developed due to the glaciers receding toward the northeast and northwest and underwent two recorded lake heights. The first was immediately post-glacial maximum in the Moreton-in-Marsh area with lake waters overflowing through the Moreton Gap (137 m OD) and incising into the Moreton moraine [13,134]. The second, at a height of 129 m OD, lasted until the Severn Valley became ice free and West Midland erratics stopped being deposited within the Wolvercote Terrace [13].

As seen in figure 14*b*, Late Wolstonian Substage glaciers (Thrussington and Oadby) advanced through the proto-Soar palaeovalley and reached their maximum extent north of Moreton-in-Marsh (SP205323) (figures 1 and 14*b*) [1,6,16,60,61,134]. The Moreton moraine ridge (figure 14*b* inset) at 145 m OD, underlain by a Triassic-rich diamicton (locally known as the Moreton Till Member), has a morphology that suggests it is a terminal moraine [13,16,134]. The moraine is overlain by the chalk-rich (Oadby) diamicton to the north (around Stretton-on-Fosse, figure 1). The moraine ridge marks the southern limit of Late Wolstonian Substage ice; this glacial event is termed here the Moreton Stadial (see appendix A).

Luminescence dating of cold-climate fluvial and glaciofluvial sediments from Meriden, Park Farm, Glebe Farm, Wolston and Seisdon, reflects the two phases of deposition across the region. The early phase at 199 ± 5 ka (figure 13) corresponds well with the early advance of the Severn Valley Welsh (Thrussington) ice at Seisdon. The later main depositional phase of extensive glaciofluvial deposition across the Warwickshire Avon and Birmingham plateau is dated to no earlier than 147 ± 3 ka (table 2; figures 1 and 13), marking the Late Wolstonian Substage glacial limit during the Moreton Stadial between the two phases. The ages correspond well to other published Late Wolstonian (=Late Saalian) Stage age samples from sites in The Netherlands, southern and northeast Germany and the eastern and southern Alps [44–46,137–140].

##### 5.2.2. Neighbouring regions

In the English East Midlands, glacial ice advancing from the northeast deposited the Wragby Till Member, equivalent to the Oadby Till Member in the English West Midlands [64]. The associated glacially derived fluvial River Trent terrace deposits of the Eagle Moor Sand and Gravel Member near Lincoln [141] are contemporaneous with the Wragby Till. White *et al.* [64] suggested that the Wragby ice in the East Midlands provides evidence for Middle Wolstonian Substage (*ca* MIS 8) glaciation in lowland Britain, based on the relationship of the glaciogenic sequence to the overlying temperate deposits. However, the correlation is questioned by Gibbard and West [130] who assign these deposits to the Ipswichian (=Eemian) Stage Interglacial (*ca* MIS 5e). Evidence for Middle Wolstonian Substage glaciation has been reported across continental Europe [142–146]. However, glaciation during this stage was significantly less extensive than that during the Late Wolstonian Substage (*ca* MIS 6) [147]. Schwenninger *et al.* [148] reported luminescence ages from East Leake, Loughborough (figures 1 and 14). This unit was correlated with an outwash gravel deposit derived from the Oadby Till (Wragby Till) ice by Bridgland *et al.* [149]. It formed during the deposition of the downstream Eagle Moor Terrace of the River Trent. This critical sequence is highly relevant because the luminescence ages from the site studied by Schwenninger *et al.* [148] place deposition to a minimum age of *ca* 178 ± 19 ka (mean of 212 ± 22, 174 ± 17 and 147 ± 17 ka). This corresponds closely to the findings in this study. It provides a clear constraint on the post-glacial deposition of the Eagle Moor deposits within the Trent catchment, and correlates to equivalent glacial advances in the English West Midlands during the Moreton Stadial (figure 14*b*).

Evidence of glaciation to the southeast in the Fenland Basin during the Late Wolstonian Substage is indicated by ice-contact, glacio-marginal delta fan deposits in proglacial lakes impounded by ice [150]. The deposits form part of a series of local glaciofluvial delta fans first identified at Tottenhill, Norfolk, that extended into proglacial lakes [54,55,149]. Ice advance is also demonstrated at Warren Hill (Three Hills), High Lodge, Lakenheath, Feltwell and Shouldham Thorpe [40,132,150,151–153] (figure 14). Here Feltwell Formation meltwater deposits, comparable to those at Tottenhill, accumulated [54]. The Fenland Basin was entirely filled by a glacier during the Late Wolstonian Substage. This ice lobe formed the eastern sector of the northeast Oadby ice in the English West Midlands and advanced down the east coast of England, entering the Fenland Basin and impounded itself against the higher ground of the Fenland margins [40,133,154]. It re-organized the rivers Nene, Ouse and Cam, and dammed several proglacial lakes within the Fenland Basin, and the Nene and Welland catchments [54,132,133,155,156]. Six luminescence ages from Shouldham Thorpe and Watlington in Norfolk and Maidscross Hill in Suffolk between 212 ± 9 and 169 ± 6 ka have been reported by Gibbard *et al.* [156] as representing ice-marginal delta and alluvial fan deposits within the Fenland Basin as ice advance during the Late Wolstonian Substage. A further seven luminescence ages from the Tottenhill sand and gravel have ages between 200 ± 33 and 125 ± 14 ka (mean of *ca* 174 ± 38 ka), constraining the maximum advance of the Fenland Basin ice to the Moreton Stadial of the Late Wolstonian Substage [157,158]. Finally, two luminescence ages from Lynford in the Wissey Valley on glacio-fluvial sands have yielded ages of 175 and 169 ka [132]. In addition, two luminescence ages within an ice-proximal depositional environment formed of outwash sands and part of the Britons Lane Sand and Gravel Member at Stiffkey in North Norfolk, have returned ages of 164 ± 11 and 141 ± 9 ka, establishing glacial margins of the Moreton Stadial ice along the North Norfolk Coast and the Fenland Basin region [154]. Reported luminescence ages at Tottenhill, Shouldham Thorpe, Watlington, Maidscross Hill and Lynford in the Fenland Basin [156–158] and Stiffkey on the North Norfolk Coast [154] support the maximum extent of the glacier during the Moreton Stadial in the region and correspond well with dating of the English West Midlands. Overall, the increasing lithostratigraphical and chronostratigraphical evidence across the English West Midlands and East Anglia strongly supports the occurrence of glaciation during the Late Wolstonian Substage (i.e during MIS 6) in the British Isles.

### 5.3. Correlation with glacial sequences in continental Europe

**Figure 15.**
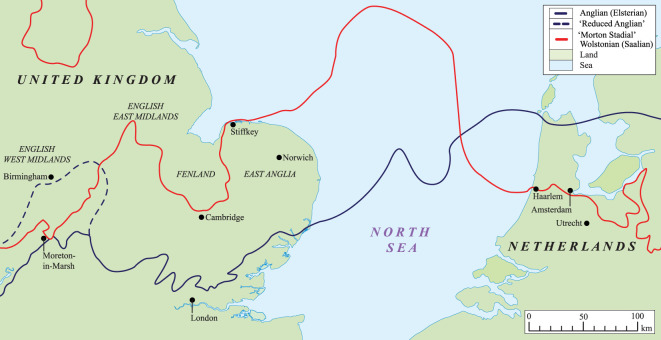
The Wolstonian Stage glacial maximum of Late Wolstonian/Saalian Substage ice limits during the Moreton/Drenthe Stadial and the preceding glacial maximum of the Anglian/Elsterian Stage across England, the North Sea, The Netherlands and western Germany. Modified from Gibson [68], Gibbard *et al.* [40,132,156] and Cartelle *et al.* [48].

Shotton [1] stated that the Pleistocene deposits of the English West Midlands were more than of local significance, suggesting that the glaciers during the Moreton Stadial were the same age as the Drenthe Stadial in The Netherlands and Germany. This correlation remains valid today. Comparisons of the glacial sequences around the North Sea are striking, with events occurring almost in parallel [40,156]. The glaciation during the Moreton Stadial, when glaciers advanced south into Midland England, is represented by the Thrussington Member and Oadby Member in the English West Midlands (phases between 199 ± 5 and 147 ± 3 ka), the Wragby Till Member in the East Midlands (178 ± 19 ka) and the Tottenhill Member (Feltwell Formation) and Britons Lane Sand and Gravel Member in the Fenland Basin (between 141 ± 9 and 212 ± 9 ka) [148,154,156–158], and has been correlated by Gibbard *et al.* [40] with glaciation during the Drenthe Stadial in The Netherlands, during the Saalian glaciation (*sensu stricto*) in northern Europe (figure 15).

The Drenthe glaciation [159] was the first major glacial advance, marking the southernmost limit of glaciation during the Saalian Stage glaciation in the central Netherlands. Here the maximum glaciation extent is represented by a complex of push-moraines and diversion of the River Rhine to a westerly course, via the Ijssel Valley, by ice advancing from the northeast [40,42,48,160–163]. Luminescence dating of the marginal outwash and ice-dammed lake sediments, associated with the maximum advance of the Drenthe Stadial ice in the central Netherlands, gives a mean age of *ca* 160–150 ka [40,163] and in northern Germany between 196 ± 19 and 153 ± 7 ka [137,138,140,163]. The general age over- and under-estimation could be explained by palaeomoisture and palaeoclimate controls on temperature forcing and water availability or down to the depositional environment preventing full bleaching [41,164]. Within the southern North Sea, a substantial proglacial lake formed during the advance of Drenthe ice towards the southwest and Moreton ice toward the southeast, converging in the North Sea basin, with the lake impounded to the south by a barrier north of the Dover Strait [40,163,165]. This is an important independent event, which caused the final breaching of the Dover Strait, with the release of major floodwaters through the English Channel during the Late Wolstonian Substage (*ca* MIS 6) [163,166,167] (figure 15).

## 6. Conclusion

A number of conclusions arise from this study:

(1) The English West Midlands contains unequivocal geological and geomorphological evidence for a substantial glaciation event that is post-Hoxnian and pre-Ipswichian stages, and occurred during the intervening Late Wolstonian Substage. The glacial sediments of the Wolston Glacigenic Formation form the regional event stratotype and accumulated during the Moreton Stadial (appendix A). The luminescence ages obtained during this study place the timing of the glaciation during the Late Wolstonian Substage in the region between 199 and 147 ka (*ca* MIS 6e–c). This timing is in advance of the global MIS 6 glacial maximum as recorded, for example, in North America where it is dated to approximately 140 ka [147].
(2) The Wolstonian Stage in the English West Midlands was characterized by the following glacial events and processes:
  (a) Extensive lowland glaciation across the region is indicated by glacial erratics whose exposure ages bracket the timing of glacial advance to between 225 ± 7 and 103 ± 13 ka.
  (b) The first phase of Late Wolstonian Substage glaciation saw ice advance into the Severn Valley from Wales and from northwest England into the Seisdon and Birmingham Plateau. The first phase of ‘Thrussington glacier’ advance has been dated by luminescence to 199 ± 5 ka, during the Moreton Stadial.
  (c) Persistence of ice in the area allowed the formation of glacial Lake Harrison across much of the region by damming of the proto-Soar and meltwater rivers from the advancing glacial ice.
  (d) The second (main) phase of Late Wolstonian Substage glaciation saw ice advance from the northeast across the Birmingham Plateau and into the Warwickshire Avon and terminate at Moreton-in-Marsh. The ‘Oadby glacier’ advance has been luminescence dated to no earlier than 147 ± 3 ka, also during the Moreton Stadial.
(3) Ice dynamics during the Moreton Stadial imply markedly different topographical controls to those of the previous, Anglian Stage. During the Moreton Stadial, glacial ice entered the area as the Welsh ‘Arenig’ ice, infilling the Severn Valley. This advance was contemporaneous with the advances of northwest Thrussington ice into the Coventry–Leicester area and the northeast Oadby ice into the proto-Soar palaeovalley. The advancing ice was stopped by the Jurassic escarpment at Daventry, Fenny Compton and Moreton-in-Marsh, which first formed a major barrier to ice advancing during the Late Wolstonian Substage. By contrast, during the Anglian Stage, glaciation in English West Midlands was of limited extent, with reported evidence of glaciation limited to Quinton [21], Nechells [15] and Gilson [7,67,68] (figures 1 and 14). At that time, the Jurassic escarpment was substantially more subdued, having not yet fully developed by periglacial weathering and erosion, suggesting that the Anglian glaciation was less influential in the region, with the majority of sediments being eroded by succeeding glaciation to limits further south during the Late Wolstonian Substage.
(4) The revised Late Wolstonian Substage glacial limits in the British Isles equate directly with those in the neighbouring northwest Europe during the Late Saalian Substage (figure 15).
(5) The drainage evolution of the English West Midlands directly relates to the Late Wolstonian Substage glaciation across the region. A proto-Soar River with the headwaters near Evesham flowed northeast through the region, probably during the Early–Middle Wolstonian Substages (*ca* MIS 10–8). During the first part of the Late Wolstonian Substage, it was independent and diachronous to the proto-Ingham River in East Anglia. The proto-Soar River was destroyed by the advancing Moreton Stadial glacier during the second part of the Late Wolstonian Substage (*ca* MIS 6).

## Data Availability

The datasets supporting this article are from the references cited or are uploaded as part of the electronic supplementary material [169]. Mapping data are from Digimap https://digimap.edina.ac.uk [74].

## Appendix A. The Moreton Stadial Event

As noted in the main text, a glacial moraine forms rising ground (145–137 m OD) around the village of Moreton-in-Marsh, Warwickshire (SP 205 323) (figures 1 and 14*b*). This moraine complex marks the known maximum extent of glaciation during the Late Wolstonian Substage within the English West Midlands. The Moreton moraine ‘drift’ deposit was first mapped by Dines [168] and identified/interpreted by Tomlinson [16]. Bishop [13] subsequently linked the deposits as the maximum southern extension of the glacial sediments which form the Wolston Glacigenic Formation of the Wolstonian Stage [1,6,11]. The Moreton moraine ‘drift’ occurs as a series of ridges for *ca* 30 km^2^ around Moreton-in-Marsh and south to Broadwell (figure 14*b*). The glaciogenic deposits were proven in a borehole to a depth of 21 m [134]. North, at Stretton-on-Fosse (SP 218382) (figure 1 and table 1, 11), Bishop [13] described a series of deposits, exposed locally, where a diamicton unit was correlated with the Oadby Till Member, which locally overlies the Moreton Till Member of Tomlinson [16] and Sumbler [134]. In the Moreton-in-Marsh area, two glacial diamicton units are recorded interdigitating with and including the local Paxford Gravel Member [16,134,168]. The stratigraphically lower, Moreton Till of the Wolston Glacigenic Formation, local to Moreton-in-Marsh, was described by Bishop [13] as a locally laminated diamicton deposited at the margin of the pro-glacial Lake Harrison. It is equivalent to the Thrussington Till Member of the Wolston Glacigenic Formation. This diamicton unit, is directly overlain by the regional Oadby Member of the Wolston Glacigenic Formation. At Stretton-on-Fosse, faulting of the upper diamicton was reported, suggesting interpretation of the landform complex as a push moraine complex [13,134]. As discussed above, the pro-glacial Lake Harrison existed through two phases. Meltwaters from the second phase incised a channel down to 128 m OD through the confining Moreton moraine ridge, forming the Moreton Gap. This channel is critical to the regional stratigraphical correlation with the Upper Thames valley fluvial sequence during the Late Wolstonian Substage. The Moreton moraine landform complex is representative of the maximum extent of regional glacial deposition (Thrussington Till and Oadby Till members) of the Wolston Glacigenic Formation [1,6,11,13,134]. Its significance in marking the Wolstonian Stage glacial maximum limits, which have been dated herein to the Late Wolstonian Substage, give the Moreton moraine regional importance. Since it represents the glacial maximum extent, the Moreton moraine-ridge complex, and its associated exposure at Stretton-on-Fosse (table 11), are therefore proposed here as the climatostratigraphic stratotype of the Moreton Stadial, the first of two stadials (the latter being that of the Pershore Stadial in Gibson [68]) that can be identified in central Britain in the Late Wolstonian Substage.

**Table 11.** Lithostratigraphical definition of the Moreton Till Member (Wolston Glacigenic Formation).

stratotype	Moreton Moraine ridge (SP 205 323) (figures 1 and 13*b*), sequence described in Dines [168], Tomlinson [16], Bishop [13], Shotton [26] and Sumbler [134].
constituent members	Paxford Gravel Member, Moreton Till Member, Oadby Till Member (all of the Wolston Glacigenic Formation).
upper boundary	Surface morphology mapped as a glacial terminal moraine, internally a glacial diamicton.
lower boundary	Base erosion into equivalent Baginton–Lillington Formation.
total thickness	Maximum proved thickness exceeds 30 m by borehole, very locally variable.
lithological	Unsorted/variable
characteristics	Paxford Gravel Member: Limestone-rich gravel with sand, silt and clay. Quartz, quartzite, siltstone, sandstone, ironstone and flint. Moreton Till Member: Basal—Clay and Silty Clay. Reddish brown with quartz, quartzite, siltstone, flint and occasional chalk. With poorly laminated local structure. Deposited as a lake clay/till. Oadby Till Member: Upper—Gravelly CLAY. Faulted. Grey with chalk, quartz-rich gravel (*Bunter pebbles*), siltstones, flint, limestone, ironstone, chert and quartzite. Deposited as a push moraine.
distribution	Forms a ridge in excess of 30 km^2^ around Moreton-in-Marsh (figure 13*b* in the main text).
